# Synthesis, Molecular Modeling and Assessment of Anticancer Activity of New Potential CYP17A1 Inhibitors

**DOI:** 10.3390/molecules31122135

**Published:** 2026-06-17

**Authors:** Michał K. Jastrzębski, Agnieszka Korga-Plewko, Magdalena Iwan, Joanna Kubik, Anna Stachniuk, Emilia Fornal, Tomasz M. Wróbel, Agnieszka A. Kaczor

**Affiliations:** 1 Doctoral School, Medical University of Lublin, 20-093 Lublin, Poland; joanna.kubik@umlub.edu.pl; 2 Department of Synthesis and Chemical Technology of Pharmaceutical Substances with Computer Modeling Laboratory, Faculty of Pharmacy, Medical University of Lublin, 4A Chodźki St., 20-093 Lublin, Poland; 3 Independent Medical Biology Unit, Medical University of Lublin, 8b Jaczewski St., 20-090 Lublin, Poland; agnieszka.korga-plewko@umlub.edu.pl; 4 Department of Toxicology, Faculty of Pharmacy, Medical University of Lublin, 8b Jaczewski St., 20-093 Lublin, Poland; magdalena.iwan@umlub.edu.pl; 5 Department of Bioanalytics, Faculty of Medical Sciences, Medical University of Lublin, 8b Jaczewski St., 20-090 Lublin, Poland; anna.stachniuk@umlub.edu.pl (A.S.); emilia.fornal@umlub.edu.pl (E.F.)

**Keywords:** castration-resistant prostate cancer (CRPC), CYP17A1 inhibitors, non-steroidal inhibitors, molecular docking, ADME profiling

## Abstract

Castration-resistant prostate cancer (CRPC) remains a significant clinical challenge due to the ability of tumor cells to undergo intratumoral androgen synthesis, a process catalyzed by the CYP17A1 enzyme. The only CYP17A1 inhibitor available in therapy, abiraterone acetate, faces significant limitations due to its steroidal structure, which causes off-target effects and generates agonistic metabolites that paradoxically stimulate the androgen receptor (AR). This study presents the development of the D2AAK1M series, a novel class of non-steroidal potential CYP17A1 inhibitors based on a pyridine–piperidine scaffold. Through biomimetic design and molecular docking, we demonstrated that these compounds have the potential to coordinate the heme iron while achieving high shape complementarity within the catalytic pocket. In silico ADME profiling indicated superior physicochemical properties compared to abiraterone, including optimal lipophilicity, enhanced water solubility, and the potential to penetrate the blood–brain barrier for targeting CNS metastases. In vitro assay results correlated with a suggested mechanism, showing preferential cytotoxicity toward androgen-dependent LNCaP cells (AR+) while sparing AR-negative lines (DU145, PC3) and healthy human fibroblasts (BJ). Our compounds present a promising starting point for further development of non-steroidal CYP17A1 inhibitors.

## 1. Introduction

Prostate cancer (PCa) is currently one of the most significant challenges in modern medicine, ranking as the second leading cause of cancer-related deaths among men in developed countries [1,2]. The development of this malignancy is a multistep process in which the androgen receptor (AR) signaling pathway plays an essential role. The AR is a ligand-dependent transcription factor belonging to the nuclear receptor superfamily. Upon activation by androgens such as testosterone or its more potent form, dihydrotestosterone (DHT), the receptor translocates to the cell nucleus. There, it binds to androgen response elements (AREs) in the promoter regions of target genes, triggering the transcription of proteins responsible for the proliferation, differentiation, and survival of prostate cells [3].

In the early stages, the tumor is typically dependent on androgens produced by the testes, allowing for effective treatment via androgen deprivation therapy (ADT). The standard of care remains blockade of the hypothalamic–pituitary–testicular axis using agonists or antagonists of luteinizing hormone-releasing hormone (LHRH), which reduces serum testosterone to castrate levels [4]. However, despite an initial clinical response, most patients progress within 24–36 months to a stage known as castration-resistant prostate cancer (CRPC). CRPC is defined as disease progression despite maintaining castrate levels of testosterone, indicating that cancer cells have adapted to an androgen-depleted environment [5].

Cancer cells develop the ability for intratumoral de novo androgen synthesis from cholesterol or adrenal precursors (especially DHEA), making the enzyme cytochrome P450 17A1 (CYP17A1) a central focus of oncological pharmacology [6].

CYP17A1 is a key enzyme in the production of glucocorticoids and sex hormones, located in the adrenal glands and gonads. In coordination with POR reductase, it catalyzes two distinct reactions: 17α-hydroxylase activity (converting pregnenolone and progesterone into their 17-hydroxy derivatives) and 17,20-lyase activity (cleaving the steroid side chain, leading to the formation of androgens) [7].

In CRPC, this enzyme also operates in the “backdoor” pathway [8]. In this alternative process, 17-hydroxyprogesterone undergoes reduction and is subsequently, due to the high efficiency of the CYP17A1 lyase, converted into DHT, bypassing testosterone [9]. This metabolic flexibility necessitates the total inhibition of CYP17A1 to effectively cut off cancer cells from androgenic stimulation.

The only selective CYP17A1 inhibitor approved by regulatory agencies (FDA, EMA) for prostate cancer treatment is abiraterone acetate [10]. Abiraterone, a steroid derivative, acts as an irreversible inhibitor that binds to the heme in the enzyme’s active center via the nitrogen atom of a pyridine substituent at the C17 position [11]. While this structure mimics natural substrates, ensuring high affinity, it also generates several pharmacological issues [7], such as a lack of selectivity between hydroxylase and lyase activities [12]. Inhibiting 17α-hydroxylase blocks cortisol synthesis, which disrupts the negative feedback loop on the hypothalamus–pituitary axis, leading to excessive ACTH secretion. Elevated ACTH levels stimulate the adrenal glands to produce mineralocorticoid precursors, such as 11-deoxycorticosterone and corticosterone, resulting in mineralocorticoid excess syndrome (MES) [13]. Clinically, this manifests as hypertension, hypokalemia, and fluid retention.

Furthermore, due to its steroidal backbone, abiraterone shows affinity for other cytochrome P450 enzymes (e.g., CYP21A2, CYP1A2, CYP2D6), potentially leading to drug interactions and hepatotoxicity [14,15]. Moreover, metabolic studies have shown that abiraterone can be converted sequentially by 3β-hydroxysteroid dehydrogenase (3β-HSD) and 5α-reductase into 3-keto-5α-abiraterone, which paradoxically acts as an androgen receptor agonist, potentially promoting tumor progression despite treatment [16].

Other compounds (Table 1), such as galeterone (TOK-001) [17] or non-steroidal orteronel (TAK-700) [18,19], were developed as an abiraterone alternative but failed in Phase III clinical trials due to not meeting overall survival endpoints or excessive toxicity. This highlights the urgent need for a new generation of non-steroidal inhibitors with improved safety profiles and higher specificity.

Recent FDA and EMA approvals include the combination of the CYP17A1 inhibitor and the PARP inhibitor *AKEEGA* [23] (Niraparib + Abiraterone Acetate), Lutetium Lu 177 Vipivotide Tetraxetan (*Pluvicto*) [24], a radioligand targeting PSMA, which received an expanded indication for use before chemotherapy in patients with mCRPC in 2025. Another option is the combination of AR blockade with DNA repair inhibition via TALZENNA + XTANDI (*Talazoparib + Enzalutamide)* [25].

In this work, we present results on the D2AAK1M series of compounds initially designed as structurally relatively distinct D2AAK1 [26] derivatives exhibiting MAO-B/AChE inhibitory activity [27,28]. Due to a lack of the assumed activity direction, PASS-2022 and PharmaExpert-2022 software [29] were used to screen for other directions of activity and potential molecular targets. PASS (Prediction of Activity Spectra for Substances) is an in silico SAR-based system that predicts the biological activity spectrum of chemical compounds from their structures, while PharmaExpert is an expert system that interprets PASS predictions to analyze mechanisms of action, drug–drug interactions, and multi-target effects within curated biological activity networks. In this screening, anticancer activity related to CYP inhibition was identified for the model compound D2AAK1M. Following preliminary studies, a series of pyridine/piperidine derivatives was designed and synthesized. Molecular docking to the CYP17A1 binding pocket was also performed, followed by in vitro studies conducted in human prostate cancer cell lines (LNCaP, DU145, PC3).

## 2. Results

### 2.1. Evolution of Non-Steroidal Inhibitors: The D2AAK1M Series

**Figure 1:**
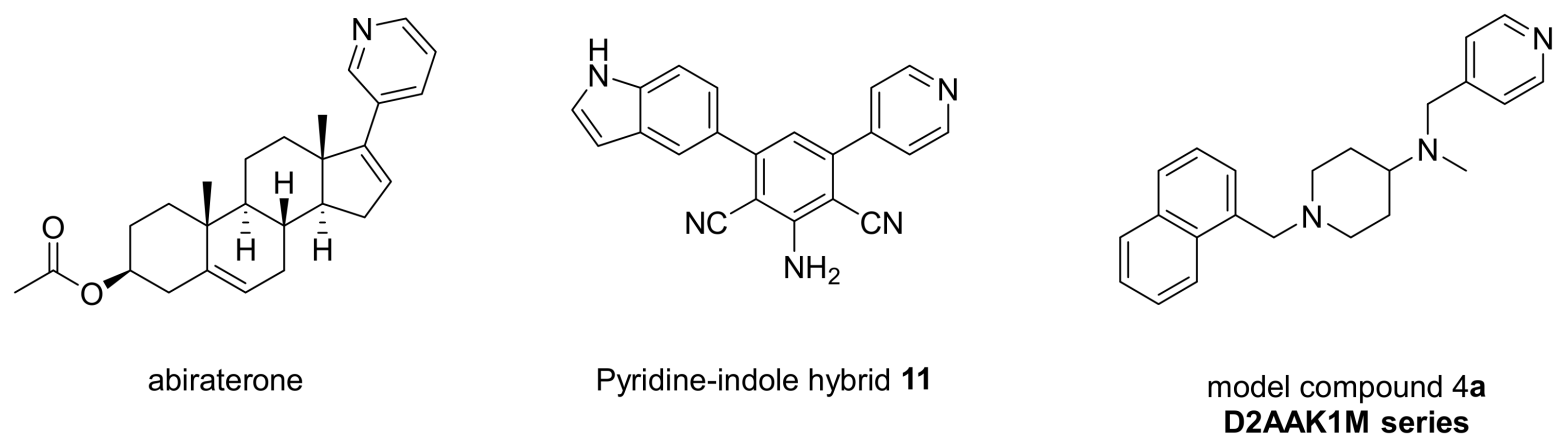
Selected CYP17A1 inhibitors.

In response to the limitations of steroidal inhibitors, research efforts have focused on non-steroidal compounds. These compounds have a lower probability of interacting with nuclear receptors and often display improved selectivity against off-target enzymes. Recent work by Wróbel et al. [14] led to the discovery and development of pyridine–indole hybrids as potent CYP17A1 inhibitors. Utilizing SAR information from known CYP17A1 inhibitors together with the structural features of abiraterone, a new series of compounds designated as the D2AAK1M series was selected. The selection strategy focused on the presence of a pyridine moiety and overall shape complementarity with the enzyme binding site, supported by PASS-based predictions of potential CYP17A1 inhibitory activity (Figure 1).

The D2AAK1M structure consists of three main modules: (1) 4-substituted pyridine scaffold that functions as the heme-binding group; the pyridine nitrogen atom forms a coordination bond with the iron ion Fe^2+^ in the heme system, which blocks access to the catalytic center; (2) a piperidine spacer allowing molecular flexibility and optimal positioning within the enzyme’s active site; (3) naphthalene moiety that serves as a hydrophobic element intended to mimic the C and D rings of natural steroids. The choice of naphthalene was dictated by its possible ability to form π–π stacking interactions with amino acid residues lining the binding pocket.

PASS (Prediction of Activity Spectra for Substances) [29] predictive analysis indicated a high probability that the D2AAK1M series compounds will not only act as potent inhibitors of steroidogenic CYP enzymes but will also exhibit direct anticancer activity. This is significant in the context of multi-directional therapeutic action, which may encompass both the inhibition of hormone production and the induction of apoptosis in cancer cells.

### 2.2. Synthesis of D2AAK1M Derivatives

The starting material for the D2AAK1M series was 4-(chloromethyl)pyridine hydrochloride or isonicotinaldehyde and their derivatives (see Section Synthesis of Compounds **4a**–**4m**). In the first step, using either reductive amination or classical *N*-alkylation, the pyridine derivatives were converted into a pyridine–piperidine intermediate. This intermediate was then deprotected by removing the -Boc group using hydrochloric acid in ethyl acetate.

**Scheme 1:**
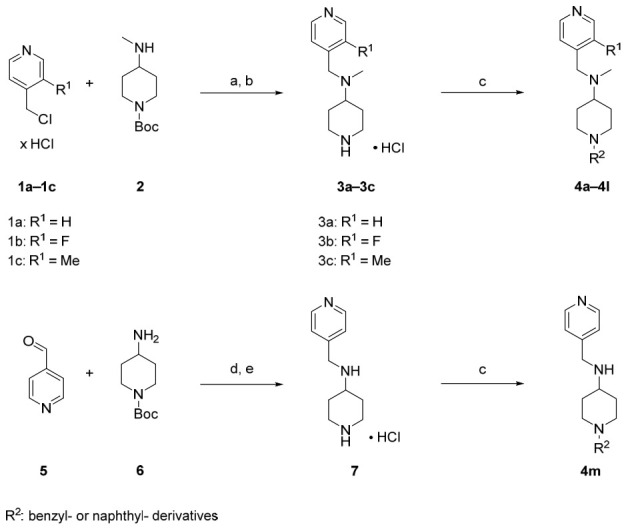
Synthesis of D2AAK1M derivatives. Reagents and conditions: (a) K_2_CO_3_, KI, ACN, 50 °C, 16 h; (b) 3M HCl/EtOAc, rt, 1 h; (c) R^2^-Cl or R^2^-Br, K_2_CO_3_, KI, DMF *or* ACN, 50 °C, 16 h; (d) NaCNBH_3_, AcOH, DCE, rt, 1 -16 h; (e) 3M HCl/EtOAc, rt, 16 h.

The resulting hydrochloride salt was employed in the synthesis of the final products. For this purpose, an *N*-alkylation reaction was performed using benzyl and naphthyl halides (Scheme 1). This procedure yielded the model compound **4a** as well as its 12 derivatives (**4b**–**4m**) of the D2AAK1M series (see also Table 2).

Alternatively, attempts were made to synthesize a piperidine ketone with the appropriate *N*-substituents and subsequently perform reductive amination with 4-aminopyridine. Unfortunately, the amination approach did not result in satisfactory yields.

### 2.3. Computational Studies

Understanding the mode of ligand binding within CYP17A1 is crucial for optimizing its biological activity and selectivity. The first crystal structures of human CYP17A1 in complex with abiraterone and galeterone, solved by DeVore and Scott [30], revealed a unique architecture of the active site. It was found that steroidal inhibitors bind in a manner that positions the molecular scaffold at an angle of approximately 60° relative to the heme plane. It is worth noting that the CYP17A1 structure exhibits significant plasticity, which was confirmed in studies with abiraterone metabolites [11].

The geometry of the resulting ligand-binding pocket complex should satisfy key assumptions, including primarily the orientation relative to the heme, the coordination of the pyridine nitrogen lone pair to the iron atom at an optimal angle maximizing binding energy, and the positioning of the aromatic group in a way that enables the formation of hydrogen bonds with the asparagine 202 (Asn202) residue located in helix F [11]. This residue is considered important for the stabilization of substrates and inhibitors, and its interaction with the 3β-hydroxyl group of abiraterone is important for its action. The design of the D2AAK1M series takes these structural aspects into account, aiming to occupy the main catalytic pocket while limiting undesirable interactions outside the active center.

#### Molecular Docking

To evaluate whether the D2AAK1M series compounds bind to CYP17A1 in a manner analogous to previously studied indole-pyridine hybrids [14], molecular docking simulations were performed using the prepared protein structure (PDB: 3RUK). Our objective was to determine if replacing the rigid, aromatic linker in the central portion of the molecule, situated between the pyridine moiety and the sterically bulk aromatic tail, with a piperidine group (which allows for conformational adaptations) would facilitate a better fit within the binding pocket and correlate with the observed in vitro parameters.

The binding pocket fit of the derivatives was also investigated in relation to the *N*-substituents attached to the piperidine fragment and the varying pyridine substituents. For the majority of the compounds, the most favorable pose positioned the molecular plane at a 60° angle relative to the heme group. The N-Fe distance indicated ligand coordination to the heme iron, maintaining a right angle between the metal atom and the nitrogen atom of the pyridine moiety (2.27–2.30 Å for various derivatives). These parameters are consistent with the model values for abiraterone, for which the coordination bond between the heme and pyridine has been structurally validated via X-ray crystallography.

Molecular docking studies conducted using SeeSAR software identified compounds **4a**, **4b**, **4c**, **4h**, and **4g** as having the optimal modeled properties. These compounds also exhibited favorable conformational parameters within the complex, including appropriate torsional angles and calculated lipophilicity.

**Figure 2:**
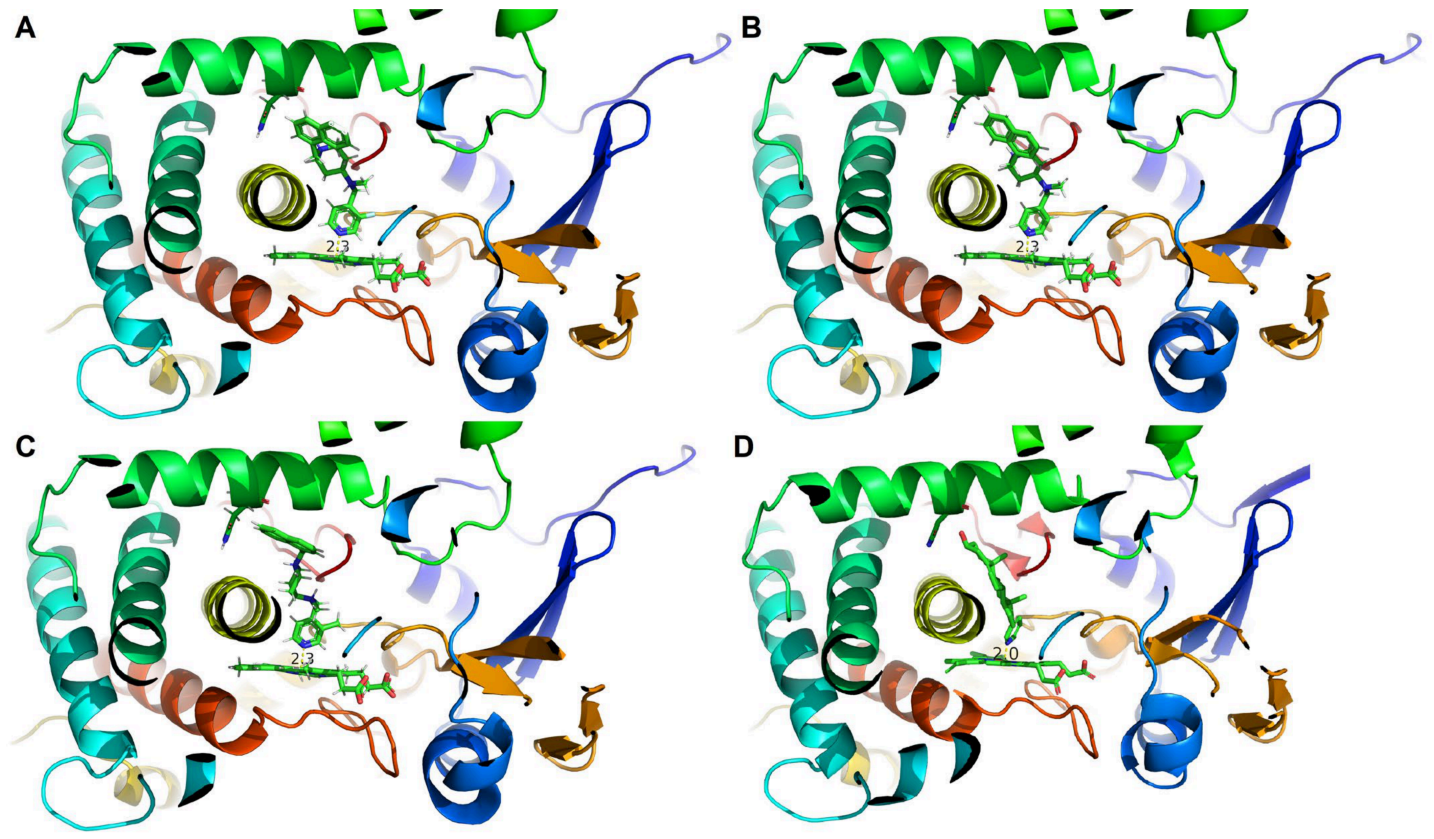
Complexes between the CYP17A1 protein (PDB: 3RUK) and ligands (**A**) **4a**, (**B**) **4b**, (**C**) **4c** and (**D**) abiraterone as a reference ligand. Ligands shown in stick representation with green carbon atoms. Protein shown in cartoon representation with rainbow coloring. The coordination bond between the heme group and the pyridine nitrogen atom is highlighted and measured (dashed yellow line). Figure prepared with PyMol.

Structurally, compounds **4a**, **4b**, and **4c** feature a naphthyl moiety, whereas compound **4l** possesses a long aliphatic linker that may mimic this moiety in certain poses. Within the analyzed complexes, no interactions beyond heme-pyridine coordination were observed; however, this may be due to the absence of functional groups with lone electron pairs in these ligands. Despite this, the compounds demonstrate high shape complementarity to the binding pocket (Figure 2, Supplementary Information SI Figure S1).

### 2.4. Evaluation of In Vitro Biological Activity

The cytotoxic activity of the tested compounds was evaluated using the MTT assay after 24 h of treatment in prostate cancer cell lines (LNCaP, DU145, PC3) and normal human fibroblasts (BJ). The obtained IC_50_ values are presented in Table 3.

**Figure 3:**
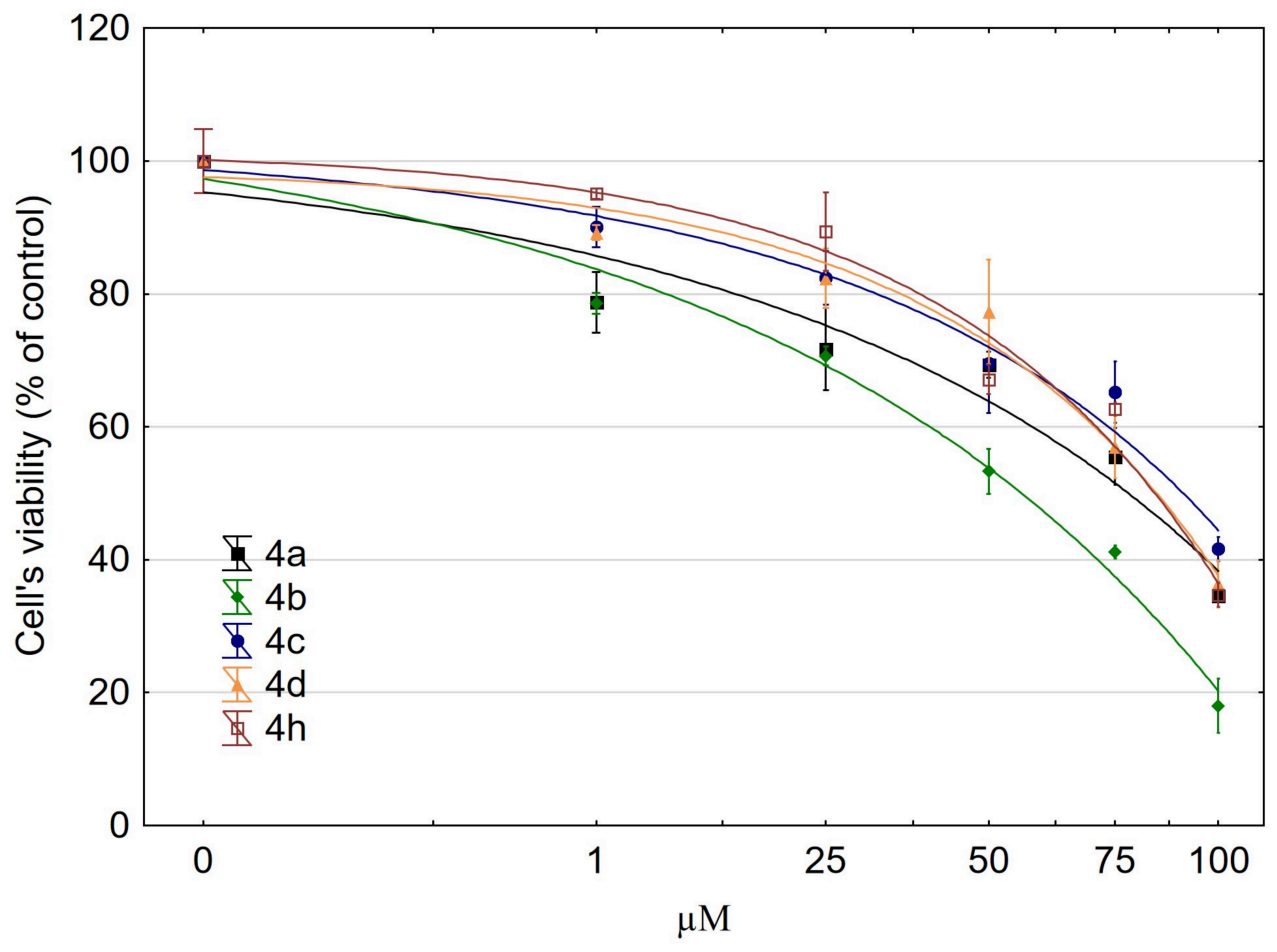
Effect of tested compounds on cell viability in LNCaP cell line after 24 h treatment, assessed by MTT test. Data are expressed as the percentage of control (mean ± SD).

Among the tested compounds, only a limited number exhibited measurable cytotoxic activity, predominantly against the LNCaP cell line. Compounds **4a**, **4b**, **4c**, **4d**, and **4h** demonstrated limited activity in LNCaP cells, with IC_50_ values ranging approximately from 62 to 94 µM. In contrast, the majority of compounds showed no significant cytotoxic effect (IC_50_ > 100 µM). Importantly, none of the tested compounds exhibited notable cytotoxicity against DU145, PC3, or normal BJ cells, indicating a lack of activity in these models under the applied conditions. This suggests a certain degree of cell-line-specific activity of the active compounds toward LNCaP cells (Figure 3).

**Figure 4:**
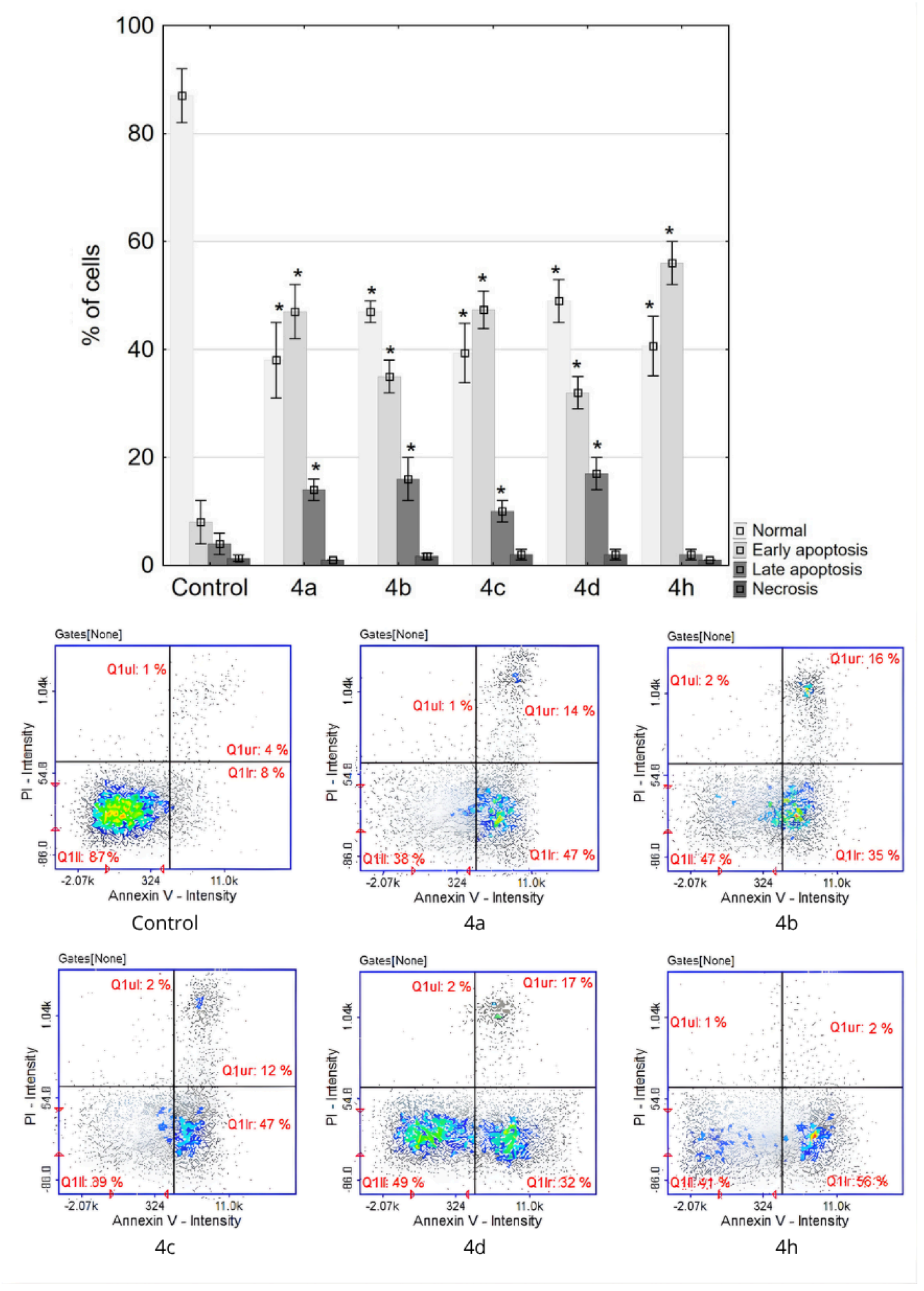
Induction of apoptosis in LNCaP cells following treatment with selected compounds at IC_50_ concentrations or with DMSO as a vehicle control for 24 h and representative histograms Cells were stained with Annexin V-FITC and propidium iodide and analyzed by image cytometry. Q1II—live cells, Q1Ir—early apoptotic cells, Q1ur—late apoptotic cells, and Q1uI—necrotic cells. * denotes *p* < 0.05. The colors indicate the density of cell events, with cooler colors representing lower densities and warmer colors representing higher densities.

Subsequently, to investigate the mechanism of cell death, apoptosis was evaluated using image cytometry for selected compounds that exhibited cytotoxic activity against LNCaP cells. An increased proportion of early apoptotic cells was observed following treatment with all tested compounds compared to the control. The highest percentage of early apoptotic cells was detected for compound **4h** (over 50%), followed by compounds **4a** and **4c** (slightly below 50%), while compounds **4b** and **4d** induced early apoptosis at approximately 30%. A population of late apoptotic cells (10–20%) was also detected for all compounds except **4h** (Figure 4).

**Figure 5:**
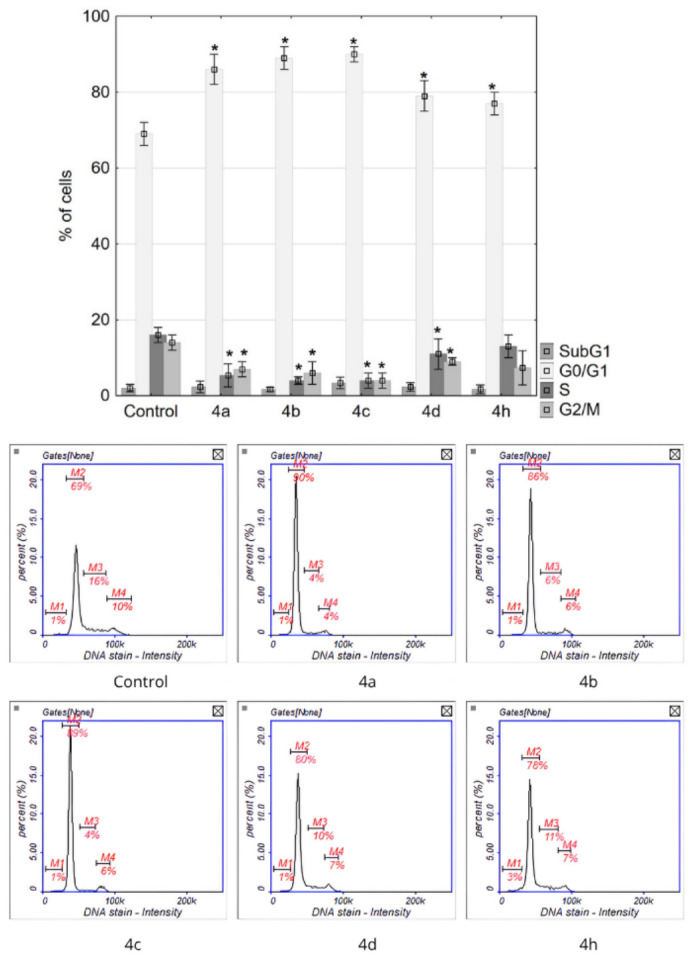
Cell cycle distribution of LNCaP cells after treatment with selected compounds at IC_50_ concentrations for 24 h or DMSO as a vehicle control and representative histograms determined by image cytometry. M1: sub-G1, M2:G0/G1, M3:S, and M4:G2/M phase. * *p* < 0.05.

Cell cycle analysis revealed an increase in the G0/G1 cell population following treatment with all tested compounds compared to the control (approximately 70%). The most pronounced effect was observed for compounds **4a**, **4b**, and **4c**, which increased the G0/G1 fraction to approximately 90%. In contrast, the remaining compounds induced a more moderate increase to around 80%. This shift was accompanied by a corresponding decrease in the S and G2/M phase populations, suggesting induction of G0/G1 cell cycle arrest (Figure 5).

The conducted studies demonstrated that the tested compounds exhibit cell-line-specific cytotoxic activity toward LNCaP prostate cancer cells, while remaining inactive in DU145 and PC3 cell lines as well as in normal BJ fibroblasts. This selectivity suggests a potential dependence of the observed biological effects on specific molecular features characteristic of LNCaP cells.

Notably, LNCaP cells are androgen receptor (AR)-positive and harbor wild-type p53, whereas DU145 and PC3 cells are AR-negative and characterized by dysfunctional or absent p53 signaling [31]. The androgen receptor plays a central role in prostate cancer progression and remains a key therapeutic target [32]. The lack of activity in DU145 and PC3 cells may therefore indicate that the mechanism of action of the active compounds is at least partially dependent on AR signaling and/or an intact p53 pathway.

Mechanistic studies performed in LNCaP cells revealed that the cytotoxic effect is associated with the induction of apoptosis, predominantly at the early stage, as well as cell cycle arrest in the G0/G1 phase. The observed accumulation of cells in G0/G1, accompanied by a reduction in S and G2/M phases, suggests inhibition of cell cycle progression and supports the involvement of regulatory pathways controlling the G1 checkpoint.

### 2.5. In Silico ADME Analysis

Modern medicinal chemistry strives to combine biological activity with an optimal ADME profile, as exemplified by compound **4a**. Although it shares a similar molecular mass with abiraterone, its unsaturated, aromatic structure based on naphthalene and pyridine provides unique properties. With a low polar surface area (19.37 Å^2^) and an absence of hydrogen bond donors, this compound exhibits excellent permeability through biological barriers.

**Figure 6:**
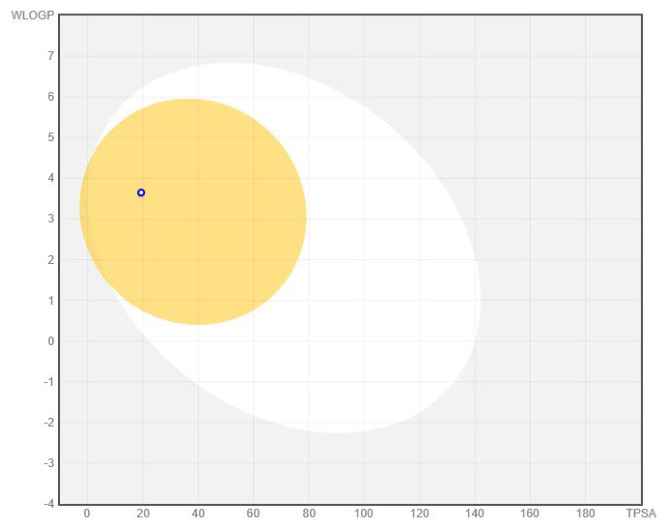
BOILED-Egg analysis for D2AAK1M compound. Yolk area—BBB. White area—HIA. Blue dot—P-gp +. Red dot—P-gp −.

The key advantage of compound **4a** is its optimal lipophilicity (3.65), which places it close to the “golden window” for oral drugs (LogD 1–3). Due to solubility an order of magnitude higher than that of abiraterone, this compound shows promise for high intestinal absorption and stable bioavailability independent of food intake. Additionally, the BOILED-Egg model indicates its ability to penetrate the blood–brain barrier (BBB), allowing it to target CNS metastases, while being a P-glycoprotein substrate limits the risk of neurotoxicity (Figure 6).

From a pharmacological perspective, the non-steroidal structure of compound **4a** eliminates the risk of forming agonistic metabolites, thereby preventing the resistance typical of abiraterone. The molecule complies with Lipinski’s and Veber’s drug-likeness criteria. Furthermore, thanks to its high synthetic accessibility (SA = 2.20) and the absence of toxicophoric alerts, it represents a safe and attractive model for optimizing oncological therapies (Table 4, Figure S1, Table S2 in Supplementary Information).

## 3. Discussion

The development of castration-resistant prostate cancer (CRPC) and the burdensome side effects of steroid-based therapies represent one of the greatest challenges in modern onco-urology. Although abiraterone effectively inhibits CYP17A1 activity, its steroidal backbone is the direct cause of its lack of selectivity, leading to mineralocorticoid excess syndrome (MES) and the formation of agonistic metabolites (e.g., 3-keto-5α-abiraterone) that stimulate the androgen receptor (AR). Furthermore, its clinical use is limited by poor pharmacokinetics, inconsistent bioavailability, and significant side effects. The results obtained for the new, non-steroidal D2AAK1M series of pyridine–piperidine hybrids demonstrate that rational drug design based on biomimetics allows for the effective overcoming of these limitations.

A key achievement in the design of the D2AAK1M series was the replacement of the rigid aliphatic linker with a piperidine system with sp^3^ carbon hybridization. Molecular docking results to the CYP17A1 crystal structure (PDB: 3RUK) confirmed the validity of this approach. The geometry of the molecules (particularly derivatives **4a**, **4b**, and **4c**) allowed for a good fit within the catalytic pocket, achieving an optimal angle of approximately 60° relative to the heme plane.

Crucially, the distance between the nitrogen atom of the pyridine ring and the iron atom was 2.27–2.30 Å, which is a textbook value [30,33] for a strong coordination bond that blocks oxygen access. Despite the lack of explicit hydrogen bonds with amino acid residues, high binding energy was achieved through nearly perfect shape complementarity and the hydrophobic interactions of the naphthyl moiety. This provides an indication that the non-steroidal core successfully mimics the natural C and D rings of steroids without carrying the burden of their structural flaws.

A common problem with oncological drugs is their low bioavailability. Abiraterone, being a highly lipophilic molecule (LogP > 5.0), requires administration on an empty stomach and is characterized by extreme absorption variability. The physicochemical profile of the model compound **4a** from the D2AAK1M series is characterized by lipophilicity close to the optimal (LogP ~ 3.65). This results in an improvement in aqueous solubility, which at the clinical stage could eliminate the food effect and facilitate dosing.

Particular attention is drawn to the modeled ability of the D2AAK1M series to penetrate the blood–brain barrier while maintaining a low polar surface area (TPSA of 19.37 Å^2^). This opens a potentially valuable therapeutic opportunity: the possibility of treating prostate cancer metastases to the central nervous system, which are a growing problem in advanced stages of the disease. The fact that the analyzed compounds are simultaneously potential P-glycoprotein substrates acts as a built-in safety valve, protecting against excessive drug accumulation in the brain and reducing the potential risk of neurotoxicity.

The results of in vitro biological studies on a cell panel provide support for the proposed mechanism of action for the new series. D2AAK1M compounds showed significant cytotoxicity (at a concentration of 100 µM) exclusively against the LNCaP line, which exhibits strong androgen receptor expression (AR+) and is highly dependent on hormonal stimulation. The lack of impact on cell signatures in androgen-independent lines (DU145 and PC3) indicates that the observed mortality of LNCaP cancer cells is not the result of a nonspecific toxic effect, but rather the result of potentially reducing intracellular androgen synthesis. Furthermore, the lack of cytotoxicity toward normal human fibroblasts (BJ line) demonstrates the high safety profile of the D2AAK1M series and a favorable therapeutic index. These findings suggest a favorable therapeutic window. Taken together, these findings indicate that the tested compounds may exert their antitumor effects through modulation of cell cycle progression and induction of apoptosis in an AR- and/or p53-dependent manner.

## 4. Materials and Methods

### 4.1. Chemistry

All reagents were obtained from commercial suppliers or were in-house available with no further purification required unless stated otherwise. NMR spectra were recorded on a Bruker AVANCE III 600 MHz spectrometer (Billerica, MA, USA) equipped with a BBO probe. Chemical shifts are presented in parts per million (δ) and are referenced to the residual proton signal in the respective solvent. Coupling constants (*J*) are reported in Hertz (Hz). High-resolution mass spectra (HRMS) were acquired on an Agilent 6550 iFunnel QTOF LC/MS mass spectrometer (Santa Clara, CA, USA), using the positive mode of electrospray ionization. Acquired spectra were processed using Agilent Mass Hunter Quantitative B. 10.1 software. NMR spectra for the synthesized compounds can be found in Supplementary Information (Figures S3–S31).

#### Synthesis of Compounds **4a**–**4m**

General procedure for the synthesis of D2AAK1M derivatives: 4-(chloromethyl)pyridine hydrochloride (1.0 eq, 1.0 mmol) **1a**, tert-butyl 4-(methylamino)piperidine-1-carboxylate (1.0 eq, 1.0 mmol) **2,** potassium carbonate (5.0 eq, 5.0 mmol), and potassium iodide (0.1 eq, 0.1 mmol) were dissolved in MeCN (50 mL) and stirred at 50 °C for 72 h, after which the solvent was evaporated, the residue was treated with water and extracted with ethyl acetate, and the combined organic layers were dried over magnesium sulfate and concentrated. The crude material was purified by column chromatography using 5–20% 7N NH_3_ in methanol/dichloromethane, and the resulting intermediate was dissolved in ethyl acetate (10 mL) and treated dropwise with 3M HCl in ethyl acetate (2.0 eq). The reaction was stirred for 16 h, cooled in an ice bath, and the resulting white hydrochloride precipitate of **3a** was filtered and dried. The alkylation reaction results in a yield of 69% and the deprotection is quantitative (>95%). Alternatively (for intermediate **7**), glacial acetic acid (1.1 eq, 1.1 mmol, 63 µL) was added to a stirred solution of tert-butyl 4-(methylamino)piperidine-1-carboxylate **6** (1.0 eq, 1.0 mmol) and isonicotinaldehyde **5** (1.0 eq, 1.0 mmol) in 1,2-dichloroethane (25 mL), the mixture was stirred for 30 min, followed by the portionwise addition of NaCNBH_3_ (1.1 eq, 1.1 mmol) and methanol (2 mL). After stirring overnight at room temperature, the reaction was quenched with saturated NaHCO_3_ solution at 0 °C, extracted with ethyl acetate (3 × 75 mL), washed with water and brine, dried over MgSO_4_, and concentrated under reduced pressure for purification via column chromatography using 5% 7N NH_3_ in methanol/dichloromethane. The purified product was then deprotected using 3M HCl in ethyl acetate (2.0 eq) for 16 h, cooled, and filtered to yield the hydrochloride salt **7**. Finally, the amine hydrochloride (1.0 eq, 0.2 mmol), an alkyl bromide or chloride (1.0 eq 0.2 mmol), and potassium carbonate (3.0 eq, 0.6 mmol) and potassium iodide (0.1 eq, 0.02 mmol) were dissolved in 5 mL of DMF and stirred vigorously at 50 °C for 16 h. The mixture was then cooled, diluted with water (50 mL), extracted with ethyl acetate (3 × 50 mL), and the organic phases were washed with water (3 × 100 mL), dried over Na_2_SO_4_, filtered, and concentrated to afford the final product as a yellowish-brown oil after column chromatography using 5–10% 7N NH_3_/MeOH in DCM.

*N-methyl-1-(naphthalen-1-ylmethyl)-N-(pyridin-4-ylmethyl)piperidin-4-amine* (**4a**)
  Substrate for the alkylation reaction: 1-(bromomethyl)naphthalene. Yield: 41% (brown oil). ^1^H NMR (600 MHz, Methanol-*d*_4_) δ 8.48–8.42 (m, 2H), 8.30–8.25 (m, 1H), 7.87 (dd, *J* = 8.0, 1.5 Hz, 1H), 7.81 (dd, *J* = 7.3, 2.2 Hz, 1H), 7.50 (dddd, *J* = 20.3, 8.1, 6.8, 1.4 Hz, 2H), 7.46–7.39 (m, 4H), 3.91 (s, 2H), 3.63 (s, 2H), 3.04 (dt, *J* = 12.0, 3.4 Hz, 2H), 2.47 (tt, *J* = 11.5, 3.8 Hz, 1H), 2.20 (s, 3H), 2.10 (td, *J* = 11.9, 2.4 Hz, 2H), 1.81 (dt, *J* = 12.7, 2.9 Hz, 2H), 1.63 (qd, *J* = 12.2, 3.9 Hz, 2H). ^13^C NMR (151 MHz, Methanol-*d*_4_) δ 150.7, 148.5, 134.0, 133.7, 132.6, 128.0, 127.7, 127.5, 125.4, 125.3, 124.7, 124.3, 124.2, 61.1, 60.2, 56.4, 53.2, 37.0, 27.4 HRMS: Calc *m*/*z* [M + H]^+^ 346.2278; Measured *m*/*z* 346.2281.
*N-((3-fluoropyridin-4-yl)methyl)-N-methyl-1-(naphthalen-1-ylmethyl)piperidin-4-amine* (**4b**)
  Substrate for the alkylation reaction: 1-(bromomethyl)naphthalene. Yield: 38% (brown oil). ^1^H NMR (600 MHz, Methanol-*d*_4_) δ 8.39 (d, *J* = 1.9 Hz, 1H), 8.35 (d, *J* = 5.0 Hz, 1H), 8.31–8.26 (m, 1H), 7.88 (dd, *J* = 8.0, 1.6 Hz, 1H), 7.85–7.79 (m, 1H), 7.60–7.41 (m, 5H), 3.94 (s, 2H), 3.73 (s, 2H), 3.07 (d, *J* = 11.2 Hz, 2H), 2.52 (tt, *J* = 11.6, 3.9 Hz, 1H), 2.25 (s, 3H), 2.19–2.11 (m, 2H), 1.84 (dt, *J* = 10.9, 3.1 Hz, 2H), 1.66 (qd, *J* = 12.2, 3.8 Hz, 2H). ^13^C NMR (151 MHz, Methanol-*d*_4_) δ 158.7 (d, *J* = 254.5 Hz), 144.9 (d, *J* = 4.9 Hz), 136.8 (d, *J* = 25.0 Hz), 136.7 (d, *J* = 13.0 Hz), 134.0, 133.7, 132.6, 128.0, 127.8, 127.5, 125.5, 125.4, 125.2, 124.7, 124.3, 61.4, 60.1, 53.1, 49.1, 37.1, 27.4. ^19^F NMR (565 MHz, Methanol-*d*_4_) δ -133.81 (d, *J* = 6.3 Hz) HRMS: Calc *m*/*z* [M + H]^+^ 364.2184; Measured *m*/*z* 364.2187.
*N-methyl-N-((3-methylpyridin-4-yl)methyl)-1-(naphthalen-1-ylmethyl)piperidin-4-amine* (**4c**)
  Substrate for the alkylation reaction: 1-(bromomethyl)naphthalene. Yield: 42% (brown oil). ^1^H NMR (600 MHz, Methanol-*d*_4_) δ 8.32–8.20 (m, 3H), 7.85 (dd, *J* = 8.0, 1.6 Hz, 1H), 7.81–7.76 (m, 1H), 7.48 (dddd, *J* = 21.3, 8.0, 6.8, 1.4 Hz, 2H), 7.44–7.37 (m, 3H), 3.91 (s, 2H), 3.60 (s, 2H), 3.08–3.00 (m, 2H), 2.49 (tt, *J* = 11.6, 3.8 Hz, 1H), 2.31 (s, 3H), 2.17 (s, 3H), 2.11 (td, *J* = 11.9, 2.4 Hz, 2H), 1.80 (dt, *J* = 12.7, 2.9 Hz, 2H), 1.65 (qd, *J* = 12.2, 3.8 Hz, 2H). ^13^C NMR (151 MHz, Methanol-*d*_4_) δ 149.2, 148.5, 146.1, 134.0, 133.7, 133.2, 132.6, 128.0, 127.8, 127.5, 125.4, 125.2, 124.7, 124.3, 123.9, 61.5, 60.1, 54.3, 53.3, 36.8, 27.3, 14.7 HRMS: Calc *m*/*z* [M + H]^+^ 360.2434; Measured *m*/*z* 360.2438.
*1-benzyl-N-methyl-N-(pyridin-4-ylmethyl)piperidin-4-amine* (**4d**)
  Substrate for the alkylation reaction: bromomethylbenzene. Yield: 56% (yellow oil). ^1^H NMR (600 MHz, Methanol-*d*_4_) δ 8.50–8.43 (m, 2H), 7.47–7.41 (m, 2H), 7.34 (d, *J* = 4.3 Hz, 4H), 7.28 (hept, *J* = 3.7 Hz, 1H), 3.67 (s, 2H), 3.53 (s, 2H), 3.00 (dq, *J* = 11.9, 2.3 Hz, 2H), 2.48 (tt, *J* = 11.6, 3.8 Hz, 1H), 2.23 (s, 3H), 2.04 (td, *J* = 12.0, 2.4 Hz, 2H), 1.86 (dt, *J* = 12.9, 3.3 Hz, 2H), 1.68 (qd, *J* = 12.2, 3.8 Hz, 2H). ^13^C NMR (151 MHz, Methanol-*d*_4_) δ 150.7, 148.5, 137.1, 129.4, 127.9, 127.0, 124.1, 62.6, 60.8, 56.4, 52.6, 37.0, 27.1 HRMS: Calc *m*/*z* [M + H]^+^ 296.2121; Measured *m*/*z* 296.2124.
*1-(4-chlorobenzyl)-N-methyl-N-(pyridin-4-ylmethyl)piperidin-4-amine* (**4e**)
  Substrate for the alkylation reaction: 1-chloro-4-(chloromethyl)benzene. Yield: 40% (yellow oil). ^1^H NMR (600 MHz, Methanol-*d*_4_) δ 8.50–8.42 (m, 2H), 7.47–7.40 (m, 2H), 7.36–7.30 (m, 4H), 3.67 (s, 2H), 3.50 (s, 2H), 2.99–2.93 (m, 2H), 2.47 (tt, *J* = 11.6, 3.8 Hz, 1H), 2.23 (s, 3H), 2.03 (td, *J* = 11.9, 2.4 Hz, 2H), 1.85 (dq, *J* = 12.5, 3.0 Hz, 2H), 1.66 (qd, *J* = 12.1, 3.8 Hz, 2H). ^13^C NMR (151 MHz, Methanol-*d*_4_) δ 150.8, 148.5, 136.2, 132.8, 130.8, 128.0, 124.1, 61.6, 60.8, 56.4, 52.6, 37.0, 27.2 HRMS: Calc *m*/*z* [M + H]^+^ 330.1732; Measured *m*/*z* 330.1736.
*1-(2,4-dichlorobenzyl)-N-methyl-N-(pyridin-4-ylmethyl)piperidin-4-amine* (**4f**)
  Substrate for the alkylation reaction: 2,4-dichloro-1-(chloromethyl)benzene. Yield: 55% (yellow oil). ^1^H NMR (600 MHz, Methanol-*d*_4_) δ 8.39–8.32 (m, 2H), 7.39 (d, *J* = 8.3 Hz, 1H), 7.36–7.31 (m, 3H), 7.22 (dd, *J* = 8.3, 2.1 Hz, 1H), 3.57 (s, 2H), 3.50 (s, 2H), 2.88 (dt, *J* = 12.7, 3.3 Hz, 2H), 2.38 (tt, *J* = 11.5, 3.8 Hz, 1H), 2.12 (s, 3H), 2.03 (td, *J* = 11.9, 2.3 Hz, 2H), 1.75 (dt, *J* = 12.5, 2.9 Hz, 2H), 1.57 (qd, *J* = 12.1, 3.9 Hz, 2H). ^13^C NMR (151 MHz, Methanol-*d*_4_) δ 150.7, 148.5, 134.9, 134.5, 133.2, 132.0, 128.7, 126.8, 124.1, 60.8, 58.1, 56.4, 52.8, 37.0, 27.4. HRMS: Calc *m*/*z* [M + H]^+^ 364.1342; Measured *m*/*z* 364.1344.
*1-(3-fluorobenzyl)-N-methyl-N-(pyridin-4-ylmethyl)piperidin-4-amine* (**4g**)
  Substrate for the alkylation reaction: 1-(chloromethyl)-3-fluorobenzene. Yield: 28% (brown oil). ^1^H NMR (600 MHz, Methanol-*d*_4_) δ 8.50–8.43 (m, 2H), 7.47–7.41 (m, 2H), 7.34 (td, *J* = 8.0, 5.9 Hz, 1H), 7.17–7.09 (m, 2H), 7.01 (td, *J* = 8.6, 2.7 Hz, 1H), 3.68 (s, 2H), 3.53 (s, 2H), 3.01–2.94 (m, 2H), 2.48 (tt, *J* = 11.5, 3.8 Hz, 1H), 2.24 (s, 3H), 2.05 (td, *J* = 11.9, 2.4 Hz, 2H), 1.86 (dt, *J* = 13.0, 2.9 Hz, 2H), 1.68 (qd, *J* = 12.2, 3.8 Hz, 2H). ^13^C NMR (151 MHz, Methanol-*d*_4_) δ 162.9 (d, *J* = 244.2 Hz), 150.7, 148.5, 140.4 (d, *J* = 7.1 Hz), 129.6 (d, *J* = 8.2 Hz), 125.0 (d, *J* = 2.9 Hz), 124.1, 115.7 (d, *J* = 21.5 Hz), 113.6 (d, *J* = 21.4 Hz), 61.9, 60.8, 56.4, 52.7, 37.0, 27.2. ^19^F NMR (565 MHz, Methanol-*d*_4_) δ-115.76 (q, *J* = 9.0 Hz). HRMS: Calc *m*/*z* [M + H]^+^ 314.2027; Measured *m*/*z* 314.2021.
*1-(3-methoxybenzyl)-N-methyl-N-(pyridin-4-ylmethyl)piperidin-4-amine* (**4h**)
  Substrate for the alkylation reaction: 1-(chloromethyl)-3-methoxybenzene. Yield: 42% (yellow oil). ^1^H NMR (600 MHz, Methanol-*d*_4_) δ 8.49–8.43 (m, 2H), 7.45–7.40 (m, 2H), 7.24 (t, *J* = 7.9 Hz, 1H), 6.93 (dd, *J* = 2.6, 1.5 Hz, 1H), 6.90 (dt, *J* = 7.5, 1.2 Hz, 1H), 6.84 (ddd, *J* = 8.3, 2.6, 1.0 Hz, 1H), 3.80 (s, 3H), 3.66 (s, 2H), 3.48 (s, 2H), 3.02–2.94 (m, 2H), 2.46 (tt, *J* = 11.6, 3.8 Hz, 1H), 2.22 (s, 3H), 2.02 (td, *J* = 12.0, 2.4 Hz, 2H), 1.84 (dt, *J* = 12.8, 2.9 Hz, 2H), 1.67 (qd, *J* = 12.2, 3.8 Hz, 2H). ^13^C NMR (151 MHz, Methanol-*d*_4_) δ 159.8, 150.8, 148.5, 138.8, 128.9, 124.1, 121.6, 114.8, 112.5, 62.6, 60.8, 56.4, 54.2, 52.7, 37.0, 27.2. HRMS: Calc *m*/*z* [M + H]^+^ 326.2227; Measured *m*/*z* 326.2231.
*N-methyl-1-phenethyl-N-(pyridin-4-ylmethyl)piperidin-4-amine* (**4i**)
  Substrate for the alkylation reaction: 2-bromoethylbenzene. Yield: 43% (dark brown oil). ^1^H NMR (600 MHz, Methanol-*d*_4_) δ 8.38–8.32 (m, 2H), 7.36–7.29 (m, 2H), 7.16 (t, *J* = 7.6 Hz, 2H), 7.12–7.04 (m, 3H), 3.57 (s, 2H), 3.04–2.98 (m, 2H), 2.75–2.66 (m, 2H), 2.51–2.45 (m, 2H), 2.38 (tt, *J* = 11.6, 3.8 Hz, 1H), 2.13 (s, 3H), 1.98 (td, *J* = 12.0, 2.4 Hz, 2H), 1.81–1.74 (m, 2H), 1.58 (qd, *J* = 12.3, 3.8 Hz, 2H). ^13^C NMR (151 MHz, Methanol-*d*_4_) δ 150.8, 148.5, 139.9, 128.3, 128.1, 125.8, 124.1, 60.7, 60.2, 56.4, 52.8, 37.0, 32.9, 27.2. HRMS: Calc *m*/*z* [M + H]^+^ 310.2278; Measured *m*/*z* 310.2278.
*1-(4-fluorophenethyl)-N-methyl-N-(pyridin-4-ylmethyl)piperidin-4-amine* (**4j**)
  Substrate for the alkylation reaction: 1-(2-chloroethyl)-4-fluorobenzene. Yield: 43% (brown oil). ^1^H NMR (600 MHz, Methanol-*d*_4_) δ 8.40–8.31 (m, 2H), 7.38–7.30 (m, 2H), 7.16–7.07 (m, 2H), 6.94–6.86 (m, 2H), 3.58 (s, 2H), 3.01 (dq, *J* = 11.1, 2.6, 1.8 Hz, 2H), 2.75–2.67 (m, 2H), 2.51–2.45 (m, 2H), 2.39 (tt, *J* = 11.5, 3.8 Hz, 1H), 2.13 (s, 3H), 2.00 (td, *J* = 12.0, 2.4 Hz, 2H), 1.79 (dq, *J* = 12.9, 2.8 Hz, 2H), 1.58 (qd, *J* = 12.2, 3.8 Hz, 2H). ^13^C NMR (151 MHz, Methanol-d4) δ 161.5 (d, *J* = 242.4 Hz), 150.8, 148.5, 135.8 (d, *J* = 3.3 Hz), 129.9 (d, *J* = 8.0 Hz), 124.1, 114.7 (d, *J* = 21.4 Hz), 60.7, 60.0, 56.4, 52.7, 37.0, 32.0, 27.2. ^19^F NMR (565 MHz, Methanol-*d*_4_) δ −119.48. HRMS: Calc *m*/*z* [M + H]^+^ 328.2184; Measured *m*/*z* 328.2189.
*N-methyl-1-(3-phenylpropyl)-N-(pyridin-4-ylmethyl)piperidin-4-amine* (**4k**)
  Substrate for the alkylation reaction: 3-bromopropylbenzene. Yield: 40% (dark brown oil). ^1^H NMR (600 MHz, Methanol-*d*_4_) δ 8.39–8.30 (m, 2H), 7.36–7.29 (m, 2H), 7.15 (t, *J* = 7.6 Hz, 2H), 7.11–7.02 (m, 3H), 3.55 (s, 2H), 2.95–2.87 (m, 2H), 2.52 (t, *J* = 7.6 Hz, 2H), 2.36 (ddd, *J* = 11.5, 7.8, 3.8 Hz, 1H), 2.30–2.23 (m, 2H), 2.11 (s, 3H), 1.94–1.85 (m, 2H), 1.74 (dq, *J* = 11.6, 8.0, 5.8 Hz, 4H), 1.54 (qd, *J* = 12.3, 3.8 Hz, 2H). ^13^C NMR (151 MHz, Methanol-*d*_4_) δ 150.8, 148.5, 141.8, 128.0, 128.0, 125.5, 124.1, 60.8, 57.9, 56.4, 52.8, 37.0, 33.4, 28.2, 27.1. HRMS: Calc *m*/*z* [M + H]^+^ 324.2434; Measured *m*/*z* 324.2438.
*N-methyl-1-(4-phenylbutyl)-N-(pyridin-4-ylmethyl)piperidin-4-amine* (**4l**)
  Substrate for the alkylation reaction: 4-bromobutylbenzene Yield: 56% (dark brown oil). ^1^H NMR (600 MHz, Methanol-*d*_4_) δ 8.37–8.31 (m, 2H), 7.34–7.28 (m, 2H), 7.14 (t, *J* = 7.6 Hz, 2H), 7.09–7.01 (m, 3H), 3.54 (s, 2H), 2.88 (dt, *J* = 12.6, 3.3 Hz, 2H), 2.52 (t, *J* = 7.5 Hz, 2H), 2.34 (tt, *J* = 11.5, 3.8 Hz, 1H), 2.27–2.20 (m, 2H), 2.10 (s, 3H), 1.85 (td, *J* = 12.0, 2.4 Hz, 2H), 1.77–1.69 (m, 2H), 1.58–1.48 (m, 4H), 1.42 (tt, *J* = 8.2, 5.6 Hz, 2H). ^13^C NMR (151 MHz, Methanol-*d*_4_) δ 150.8, 148.5, 142.2, 128.1, 127.9, 125.4, 124.1, 60.8, 58.2, 56.4, 52.8, 37.0, 35.3, 29.3, 27.1, 25.9. HRMS: Calc *m*/*z* [M + H]^+^ 338.2591; Measured *m*/*z* 338.2595.
*1-(4-methoxybenzyl)-N-(pyridin-4-ylmethyl)piperidin-4-amine* (**4m**)
  Substrate for the alkylation reaction: 1-(chloromethyl)-4-methoxybenzene. Yield: 33% (yellow oil). ^1^H NMR (600 MHz, Methanol-*d*_4_) δ 8.50–8.45 (m, 2H), 7.46–7.43 (m, 2H), 7.24 (d, *J* = 8.6 Hz, 2H), 6.91–6.87 (m, 2H), 3.85 (s, 2H), 3.79 (s, 3H), 3.45 (s, 2H), 2.95–2.87 (m, 2H), 2.48 (tt, *J* = 10.8, 4.0 Hz, 1H), 2.02 (td, *J* = 12.1, 2.5 Hz, 2H), 1.96–1.89 (m, 2H), 1.52–1.42 (m, 2H). ^13^C NMR (151 MHz, Methanol-*d*_4_) δ 159.2, 150.7, 148.6, 130.6, 128.9, 123.6, 113.2, 61.9, 54.3, 54.0, 51.7, 48.6, 31.1 HRMS: Calc *m*/*z* [M + H]^+^ 312.2070; Measured *m*/*z* 312.2077.

### 4.2. In Vitro Assays

#### 4.2.1. Cell Lines and Culture Conditions

Human prostate cancer cell lines LNCaP, DU145, and PC3, as well as normal human fibroblasts BJ, were used in this study. Cells were obtained from American Type Culture Collection (ATCC, Manassas, VA, USA) and cultured under standard conditions at 37 °C in a humidified atmosphere containing 5% CO_2_.

LNCaP cells were maintained in RPMI-1640 medium (Corning, Corning, NY, USA), while DU145, PC3, and BJ cells were cultured in Dulbecco’s Modified Eagle Medium (DMEM, Corning, USA), all supplemented with 10% fetal bovine serum (FBS, Corning, USA) and 100 U/mL penicillin, 100 μg/mL streptomycin (Sigma-Aldrich, Darmstadt, Germany). Cells were passaged upon reaching 70–80% confluence using trypsin–EDTA solution.

#### 4.2.2. Tested Compounds and Treatment Conditions

Tested compounds were dissolved in DMSO (Avantor, Gliwice, Poland) to prepare stock solutions and further diluted in culture medium to obtain the desired working concentrations. The final concentration of the solvent in the culture medium did not exceed 0.5% *v*/*v*. Cells were seeded at a density of 2 × 10^4^ cells/well and allowed to grow until they reached approximately 70–80% confluence. At this stage, the culture medium was replaced with fresh medium containing the tested compounds at the indicated concentrations. Control cells were treated with vehicle only. Cells were incubated with the compounds for 24 h.

#### 4.2.3. Cytotoxicity (MTT Assay)

Cell viability was assessed using the MTT assay. Briefly, cells were seeded in 96-well plates and allowed to reach approximately 70–80% confluence before treatment. Cells were then exposed to tested compounds for 24 h. Following treatment, MTT reagent (Invitrogen, Carlsbad, CA, USA) in final concentration of 0.5 mg/mL was added to each well and incubated for 24 h at 37 °C. After incubation, the medium was removed, and the resulting formazan crystals were dissolved in DMSO. Absorbance was measured at 570 nm using a PowerWave microplate reader (Biotek Instruments, Winooski, VT, USA). All experiments were performed in triplicate and repeated three times. Cell viability was expressed as a percentage relative to control cells. IC_50_ values were determined using the AAT Bioquest IC_50_ calculator (“Quest Graph™ IC50 Calculator.” AAT Bioquest, Inc., Pleasanton, CA, USA, 25 April 2026, https://www.aatbio.com/tools/ic50-calculator).

#### 4.2.4. Apoptosis Assay

Apoptosis detection was evaluated using the Annexin V assay with the NucleoCounter^®^ NC-3000™ system (Chemometec, Lillerød, Denmark). Cells were seeded and cultured until they reached approximately 70–80% confluence before treatment. Subsequently, cells were treated with selected compounds at IC_50_ concentrations for 24 h, harvested, and stained with Annexin V-FITC and propidium iodide (PI) according to the manufacturer’s instructions.

Stained cells were analyzed using the NC-3000™ system. The percentages of viable, early apoptotic, late apoptotic, and necrotic cells were determined using NucleoView™ software. All experiments were performed in at least three independent biological replicates.

#### 4.2.5. Cell Cycle Analysis

Cell cycle distribution was analyzed using image cytometry with the NucleoCounter^®^ NC-3000™ system (Chemometec, Denmark). Cells were seeded in 6-well plates and cultured until they reached approximately 70–80% confluence prior to treatment. Cells were then treated with selected compounds at concentrations corresponding to IC_50_ values for 24 h. After treatment, the cells were harvested using a trypsin-EDTA solution (Corning, USA), washed with PBS (Corning, USA), and stained with DAPI staining solution according to the manufacturer’s protocol. The cell suspensions were loaded into an 8-chamber NC slide and DNA content was measured, and the percentage of cells in SubG1, G0/G1, S, and G2/M phases was determined using NucleoView™ software. All experiments were performed in at least three independent biological replicates.

### 4.3. Screening for Molecular Targets of D2AAK1M Compound

To evaluate the therapeutic potential and pharmacological profile of D2AAK1M derivative **4a**, the PASS-2022 (Prediction of Activity Spectra for Substances) and PharmaExpert-2022 software were utilized [29]. PASS was employed to predict the biological activity spectrum of the compound based on 2D molecular similarity, providing insights into its potential mechanisms of action and therapeutic applications.

### 4.4. In Silico ADME Prediction

Physicochemical properties, pharmacokinetic profiles, and drug-likeness of the synthesized D2AAK1M series were evaluated using the SwissADME web tool (Swiss Institute of Bioinformatics, http://www.swissadme.ch). The molecular structures were converted into Simplified Molecular Input Line Entry System (SMILES) format and submitted to the server for computation. The analysis included the assessment of lipophilicity (expressed as Consensus Log P_o/w_), water solubility (ESOL and SILICOS-IT methods), and topological polar surface area (TPSA). Pharmacokinetic parameters, such as gastrointestinal (GI) absorption, blood–brain barrier (BBB) permeation, and P-glycoprotein (P-gp) substrate status, were predicted using the BOILED-Egg model. Drug-likeness was assessed based on Lipinski, Veber, and Egan rules, and synthetic accessibility (SA Score) was calculated to evaluate the complexity of the chemical structures.

### 4.5. Molecular Docking

Protein was prepared using PyMol v3.1.6.1. Modeling of ligands and molecular docking were performed using SeeSAR software (SeeSAR version 12.1.0—BioSolveIT GmbH, St. Augustin, Germany). The grid was generated based on co-crystalized inhibitors in X-ray structures of Human Cytochrome P450 CYP17A1 (PDB ID: 3RUK), i.e., abiraterone complex. Up to 10 poses were generated in case of each ligand-receptor complex. The final poses were selected based on the affinity range prediction and visual inspection. SeeSAR software and PyMol v3.1.6.1. tools were used for the visualization of molecular docking results.

### 4.6. Statistical Analysis

Statistical analysis was performed using STATISTICA 13 software (StatSoft, Kraków, Poland). The data were calculated as mean ± SD. Student’s *t*-test was used for the comparison of apoptosis detection and cell cycle analysis results between control and treated cells. A *p*-value of less than 0.05 was considered statistically significant.

## 5. Conclusions

The results obtained for the D2AAK1M series support the notion that potential CYP17A1 inhibition may also be achieved using non-steroidal scaffolds, which may offer a route to circumvent some of the structural limitations associated with abiraterone. Due to their rational fit within the heme pocket and an optimal ADME profile, compounds such as **4a**, **4b** and **4c** represent a promising starting point for further elaboration and more detailed in vitro studies on a new generation of drugs for castration-resistant prostate cancer. The continuation of the project should propose further structural modifications that, while retaining the aromatic naphthalene fragment, introduce a functional group capable of forming hydrogen bonds with Asn202. Further studies are required to elucidate the precise molecular targets involved in this activity. In particular, investigation of AR signaling modulation, expression of p53-related proteins, and downstream regulators of the G1/S transition (e.g., cyclins and CDKs) would help to better understand the mechanism of action. Although the cytotoxic effects were limited, the ability of the compounds to induce early apoptosis and cell cycle arrest suggests that they may serve as promising lead structures for further structural optimization. Even relatively low activity at this stage may be advantageous if accompanied by selectivity, as it provides a basis for rational design of more potent derivatives. Additionally, future work should aim to investigate the selectivity of interactions across other CYP enzymes and optimize the activity to determine and minimize the necessary dose required to achieve a therapeutic effect in both in vitro and in vivo models.

## Figures and Tables

**Table 1:** Comparison of CYP17A1 Inhibitors.

Compound Name	Structure Type	Heme-Binding Group	Clinical Status	Main Drawbacks	Reference
Ketoconazole	Non-steroidal	Imidazole	Off-label	Low selectivity, hepatotoxicity	[20]
Abiraterone acetate	Steroidal	Pyridine	Approved (FDA/EMA)	Requires steroid co-administration, MES	[21]
Orteronel (TAK-700)	Non-steroidal	Imidazole	Discontinued	No advantage over standards in Phase III (despite benefits in rPFS)	[18,19]
Galeterone (TOK-001)	Steroidal	Benzimidazole	Discontinued	Pharmacokinetic issues	
Seviteronel (VT-464)	Non-steroidal	Triazole	Discontinued	Toxicity, lack of efficacy	[22]

**Table 2:** The obtained D2AAK1M derivatives.

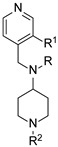			
Name	R^1^	R^2^	R
**4a** (model)	H	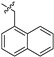	Me
**4b**	F	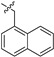	Me
**4c**	Me	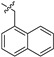	Me
**4d**	H	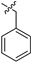	Me
**4e**	H	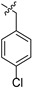	Me
**4f**	H	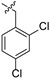	Me
**4g**	H	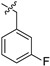	Me
**4h**	H	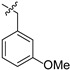	Me
**4i**	H	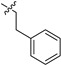	Me
**4j**	H	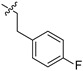	Me
**4k**	H	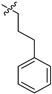	Me
**4l**	H	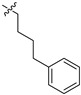	Me
**4m**	H	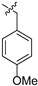	H

**Table 3:** IC_50_ values of the tested compounds in human prostate cancer cell lines (LNCaP, DU145, PC3) and normal human fibroblasts (BJ) after 24 h treatment based on MTT test results. Values represent the mean ± standard deviation from at least three independent experiments. Compounds with IC_50_ values > 100 µM were considered inactive under the tested conditions.

	IC_50_ ± SD [µM]			
Compound	LNCaP	DU145	PC3	BJ
**4a**	85 ± 11.43	>100	>100	>100
**4b**	62 ± 7.05	>100	>100	>100
**4c**	94 ± 9.67	>100	>100	>100
**4d**	82 ± 5.25	>100	>100	>100
**4e**	>100	>100	>100	>100
**4f**	>100	>100	>100	>100
**4g**	>100	>100	>100	>100
**4h**	81 ± 7.87	>100	>100	>100
**4i**	>100	>100	>100	>100
**4j**	>100	>100	>100	>100
**4k**	>100	>100	>100	>100
**4l**	>100	>100	>100	>100
**4m**	>100	>100	>100	>100

**Table 4:** Comparison of modeled ADME physicochemical data.

Lipophilicity LogP_o/w_	3.65
Topological Polar Surface Area (TPSA)	19.37 Å^2^
Water solubility	Moderately soluble
Gastrointestinal (GI) absorption	High
Blood–brain barrier permeation	Yes
P-glycoprotein (P-gp) interactions	Yes
CYP enzyme inhibition	Yes
Lipinski’s Rule of Five	Yes
Synthetic Accessibility (SA) Score	2.20
Fraction Csp^3^	0.35

## Data Availability

The data that support the findings of this study are available in the Supplementary Materials of this article. The data are also available at https://doi.org/10.18150/KGTDSX.

## Supplementary Materials

The following supporting information can be downloaded at: https://www.mdpi.com/article/10.3390/molecules31122135/s1, Table S1: Viability of LNCaP cells, AR-negative lines (DU145 and PC3) and on BJ fibroblasts (normal human cells) at a concentration of 100 µM [%]; Table S2: Possible toxic and adverse effects at P_a_ ≥ P_i_; Figure S1: Complexes between the CYP17A1 protein (PDB: 3RUK) and ligands (top) **4a**, (medium) **4b**, and (bottom) **4c**, proposed by SeeSAR. The coordination bond between the heme group and the pyridine nitrogen atom is highlighted, along with two amino acid residues: Asn202 and Cys442. The pocket itself is composed of the following residues: Ala105, Ala113, Phe114, Ile198, Tyr201, Asn202 (a key residue proximal to the aromatic naphthalene fragment), Glu203, Ile205, Ile206, Leu209, Leu214, His235, Arg239, Gly297, Asp298, Ile299, Phe300, Gly301, Ala302, Gly303, Glu305, Thr306, Val366, Ala367, Leu370, Ile371, Cys442 (the residue coordinating the heme), Val482, and Val483; Figure S2: Modeled ADME physicochemical data (full SwissADME report); Figures S3–S31: ^1^H, ^19^F and ^13^C NMR spectra of the investigated compounds.

## Abbreviations

The following abbreviations are used in this manuscript:

3β-HSD	3β-hydroxysteroid dehydrogenase
AChE	Acetylcholinesterase
ACN	Acetonitrile
ADME	Absorption, Distribution, Metabolism, and Excretion
ADT	Androgen deprivation therapy.
AR	Androgen Receptor
AR-	androgen-independent
AREs	Androgen response elements
Asn	Asparagine
BBB	Blood–brain barrier
BJ	Healthy human fibroblasts
Boc	tert-Butyloxycarbonyl group
CNS	Central nervous system
CRPC	Castration-resistant prostate cancer
CYP17A1	Enzyme cytochrome P450 17A1
Cys	Cysteine
DHT	Dihydrotestosterone
DMF	Dimethylformamide
DU145	Human prostate cancer cell line
EtOAc	Ethyl acetate
GI	Gastrointestinal
HIA	High passive absorption in the gastrointestinal tract
LHRH	Luteinizing hormone-releasing hormone
LNCaP cells	Lymph Node Carcinoma of the Prostate cells
LogP	Octanol-water partition coefficient
MAO-B	Monoamine oxidase B
Me	Methyl group
MES	Mmineralocorticoid excess syndrome
MTT	(3-[4,5-dimethylthiazol-2-yl]-2,5 diphenyl tetrazolium bromide) assay
PCa	Prostate cancer
P-gp	P-glycoprotein
rPFS	radiographic Progression-Free Survival
SA	Synthetic Accessibility
TPSA	Topological Polar Surface Area

## References

[1] Sung H., Ferlay J., Siegel R.L., Laversanne M., Soerjomataram I., Jemal A., Bray F. (2021). Global Cancer Statistics 2020: GLOBOCAN Estimates of Incidence and Mortality Worldwide for 36 Cancers in 185 Countries. CA Cancer J. Clin..

[2] Siegel R.L., Kratzer T.B., Wagle N.S., Sung H., Jemal A. (2026). Cancer Statistics, 2026. CA Cancer J. Clin..

[3] Tan M.H.E., Li J., Xu H.E., Melcher K., Yong E. (2015). Androgen Receptor: Structure, Role in Prostate Cancer and Drug Discovery. Acta Pharmacol. Sin..

[4] Zhao J., Chen J., Sun G., Shen P., Zeng H. (2024). Luteinizing Hormone-Releasing Hormone Receptor Agonists and Antagonists in Prostate Cancer: Effects on Long-Term Survival and Combined Therapy with next-Generation Hormonal Agents. Cancer Biol. Med..

[5] Karantanos T., Evans C.P., Tombal B., Thompson T.C., Montironi R., Isaacs W.B. (2015). Understanding the Mechanisms of Androgen Deprivation Resistance in Prostate Cancer at the Molecular Level. Eur. Urol..

[6] Montgomery R.B., Mostaghel E.A., Vessella R., Hess D.L., Kalhorn T.F., Higano C.S., True L.D., Nelson P.S. (2008). Maintenance of Intratumoral Androgens in Metastatic Prostate Cancer: A Mechanism for Castration-Resistant Tumor Growth. Cancer Res..

[7] Wróbel T.M., Jørgensen F.S., Pandey A.V., Grudzińska A., Sharma K., Yakubu J., Björkling F. (2023). Non-Steroidal CYP17A1 Inhibitors: Discovery and Assessment. J. Med. Chem..

[8] Fiandalo M.V., Wilton J., Mohler J.L. (2014). Roles for the Backdoor Pathway of Androgen Metabolism in Prostate Cancer Response to Castration and Drug Treatment. Int. J. Biol. Sci..

[9] Lee H.G., Kim C.J. (2022). Classic and Backdoor Pathways of Androgen Biosynthesis in Human Sexual Development. Ann. Pediatr. Endocrinol. Metab..

[10] Rehman Y., Rosenberg J.E. (2012). Abiraterone Acetate: Oral Androgen Biosynthesis Inhibitor for Treatment of Castration-Resistant Prostate Cancer. Drug Des. Devel. Ther..

[11] Petrunak E.M., Bart A.G., Peng H.-M., Auchus R.J., Scott E.E. (2023). Human Cytochrome P450 17A1 Structures with Metabolites of Prostate Cancer Drug Abiraterone Reveal Substrate-Binding Plasticity and a Second Binding Site. J. Biol. Chem..

[12] Bird I.M., Abbott D.H. (2016). The Hunt for a Selective 17,20 Lyase Inhibitor; Learning Lessons from Nature. J. Steroid Biochem. Mol. Biol..

[13] Ferraldeschi R., Sharifi N., Auchus R.J., Attard G. (2013). Molecular Pathways: Inhibiting Steroid Biosynthesis in Prostate Cancer. Clin. Cancer Res..

[14] Wróbel T.M., Grudzińska A., Yakubu J., du Toit T., Sharma K., Harrington J.C., Björkling F., Jørgensen F.S., Pandey A.V. (2025). Pyridine Indole Hybrids as Novel Potent CYP17A1 Inhibitors. J. Enzym. Inhib. Med. Chem..

[15] Obst J.K., Sadar M.D. (2016). Directing Abiraterone Metabolism: Balancing the Scales between Clinical Relevance and Experimental Observation. Transl. Cancer Res..

[16] Li Z., Alyamani M., Li J., Rogacki K., Abazeed M., Upadhyay S.K., Balk S.P., Taplin M.-E., Auchus R.J., Sharifi N. (2016). Redirecting Abiraterone Metabolism to Fine-Tune Prostate Cancer Anti-Androgen Therapy. Nature.

[17] Bastos D.A., Antonarakis E.S. (2016). Galeterone for the Treatment of Advanced Prostate Cancer: The Evidence to Date. Drug Des. Devel. Ther..

[18] Van Hook K., Huang T., Alumkal J.J. (2014). Orteronel for the Treatment of Prostate Cancer. Future Oncol..

[19] Fizazi K., Jones R., Oudard S., Efstathiou E., Saad F., de Wit R., De Bono J., Cruz F.M., Fountzilas G., Ulys A. (2015). Phase III, Randomized, Double-Blind, Multicenter Trial Comparing Orteronel (TAK-700) plus Prednisone with Placebo plus Prednisone in Patients with Metastatic Castration-Resistant Prostate Cancer That Has Progressed during or after Docetaxel-Based Therapy: ELM-PC 5. J. Clin. Oncol..

[20] Harris K.A., Weinberg V., Bok R.A., Kakefuda M., Small E.J. (2002). Low Dose Ketoconazole With Replacement Doses of Hydrocortisone in Patients With Progressive Androgen Independent Prostate Cancer. J. Urol..

[21] de Bono J.S., Logothetis C.J., Molina A., Fizazi K., North S., Chu L., Chi K.N., Jones R.J., Goodman O.B., Saad F. (2011). Abiraterone and Increased Survival in Metastatic Prostate Cancer. N. Engl. J. Med..

[22] Madan R.A., Schmidt K.T., Karzai F., Peer C.J., Cordes L.M., Chau C.H., Steinberg S.M., Owens H., Eisner J., Moore W.R. (2020). Phase 2 Study of Seviteronel (INO-464) in Patients With Metastatic Castration-Resistant Prostate Cancer After Enzalutamide Treatment. Clin. Genitourin. Cancer.

[23] Janssen Marks First Approval Worldwide for AKEEGA^®^ (Niraparib and Abiraterone Acetate Dual Action Tablet) with EC Authorisation for the Treatment of Patients with Metastatic Castration Resistant Prostate Cancer with BRCA1/2 Mutation. https://s203.q4cdn.com/636242992/files/doc_news/2023/04/AKEEGA-Niraparib-and-Abiraterone-Acetate-Dual-Action-Tablet-approval-press-release-1.pdf.

[24] Keam S.J. (2022). Lutetium Lu 177 Vipivotide Tetraxetan: First Approval. Mol. Diagn. Ther..

[25] Lukan C., Suh K. (2026). Economic Evaluation of Talazoparib and Enzalutamide Compared to Enzalutamide Monotherapy in HRRm Metastatic Castration-Resistant Prostate Cancer. J. Am. Pharm. Assoc..

[26] Kaczor A.A., Targowska-Duda K.M., Budzyńska B., Biała G., Silva A.G., Castro M. (2016). In Vitro, Molecular Modeling and Behavioral Studies of 3-{[4-(5-Methoxy-1H-Indol-3-Yl)-1,2,3,6-Tetrahydropyridin-1-Yl]Methyl}-1,2-Dihydroquinolin-2-One (D2AAK1) as a Potential Antipsychotic. Neurochem. Int..

[27] Jastrzębski M.K., Wójcik P., Mudgal A., Woźniak S., Targowska-Duda K.M., Wronikowska-Denysiuk O., Michalak A., Jeleniewicz W., Karcz T., Raczek J. (2025). Unveiling the Therapeutic Potential of D2AAK1 and Its Derivatives: Mechanistic Insights and Applications in Neurodegenerative Disease Treatment. J. Neurochem..

[28] Asim A., Jastrzębski M.K., Kaczor A.A. (2025). Dual Inhibitors of Acetylcholinesterase and Monoamine Oxidase-B for the Treatment of Alzheimer’s Disease. Molecules.

[29] Poroikov V.V., Filimonov D.A., Borodina Y.V., Lagunin A.A., Kos A. (2000). Robustness of Biological Activity Spectra Predicting by Computer Program PASS for Noncongeneric Sets of Chemical Compounds. J. Chem. Inf. Comput. Sci..

[30] DeVore N.M., Scott E.E. (2012). Structures of Cytochrome P450 17A1 with Prostate Cancer Drugs Abiraterone and TOK-001. Nature.

[31] Saranyutanon S., Deshmukh S.K., Dasgupta S., Pai S., Singh S., Singh A.P. (2020). Cellular and Molecular Progression of Prostate Cancer: Models for Basic and Preclinical Research. Cancers.

[32] Quistini A., Chierigo F., Fallara G., Depalma M., Tozzi M., Maggi M., Jannello L.M.I., Pellegrino F., Mantica G., Terracciano D. (2025). Androgen Receptor Signalling in Prostate Cancer: Mechanisms of Resistance to Endocrine Therapies. Res. Rep. Urol..

[33] Fehl C., Vogt C.D., Yadav R., Li K., Scott E.E., Aubé J. (2018). Structure-Based Design of Inhibitors with Improved Selectivity for Steroidogenic Cytochrome P450 17A1 over Cytochrome P450 21A2. J. Med. Chem..

