# Supplementary material for: Synthesis, Molecular Modeling and Assessment of Anticancer Activity of New Potential CYP17A1 Inhibitors
Source: Molecules. 2026 Jun 17;31(12):2135. doi: 10.3390/molecules31122135 (PMC13306170; doi:10.3390/molecules31122135)
Supplement: Supplementary file 1 [file molecules-31-02135-s001.zip › molecules-4322903-supplementary.pdf]

Supplementary Information

Synthesis, Molecular Modeling and Assessment of Anticancer Activity of New Potential CYP17A1 Inhibitors

Michał K. Jastrzębski [1,2,*], Agnieszka Korga-Plewko [3], Magdalena Iwan [4], Joanna Kubik [1,3], Anna Stachniuk [5], Emilia Fornal [5], Tomasz M. Wróbel [2,*] and Agnieszka A. Kaczor [2,*]

[1] Doctoral School, Medical University of Lublin, 20-093 Lublin, Poland; joanna.kubik@umlub.edu.pl
[2] Department of Synthesis and Chemical Technology of Pharmaceutical Substances with Computer Modeling Laboratory, Faculty of Pharmacy, Medical University of Lublin, 4A Chodźki St., 20-093 Lublin, Poland
[3] Independent Medical Biology Unit, Medical University of Lublin, 8b Jaczewski St., 20-090 Lublin, Poland; agnieszka.korga-plewko@umlub.edu.pl
[4] Department of Toxicology, Faculty of Pharmacy, Medical University of Lublin, 8b Jaczewski St., 20-093 Lublin, Poland; magdalena.iwan@umlub.edu.pl
[5] Department of Bioanalytics, Faculty of Medical Sciences, Medical University of Lublin, 8b Jaczewski St., 20-090 Lublin, Poland; anna.stachniuk@umlub.edu.pl (A.S.); emilia.fornal@umlub.edu.pl (E.F.)
* Correspondence: michal.jastrz1998@gmail.com (M.K.J.); tomasz.wrobel@umlub.edu.pl (T.M.W.); agnieszka.kaczor@umlub.edu.pl (A.A.K.)

Supplementary Tables S1 and S2
Supplementary Figures S1-S2
^{1}H, ^{19}F and ^{13}C NMR spectra of the investigated compounds (Figures S3-S31).

Table S1. Viability of LNCaP cells, AR-negative lines (DU-145 and PC-3) and on BJ fibroblasts (normal human cells) at a concentration of 100 μM [%].

**Compound**	**35**	**36**	**37**	**69**	**73**	**81**	**83**
LNCaP 24h	16.34	24.20	23.80	42.06	62.94	58.21	61.97
LNCaP 48h	16.17	12.30	27.45	55.06	86.97	46.91	63.68
DU145 24h	107.61	99.12	101.02	100.37	88.20	84.11	77.02
DU145 48h	92.15	80.55	84.08	114.33	91.85	83.28	78.59
PC3 24h	84.13	91.76	91.61	75.74	83.31	75.12	82.36
PC3 48h	81.79	78.84	76.19	68.46	83.57	75.53	58.21
BJ 24h	105	110	107	100	103	104	99
BJ 48h	106	115	116	96	104	95	109

Table S1 (continuation). Viability of LNCaP cells, AR-negative lines (DU-145 and PC-3) and on BJ fibroblasts (normal human cells) at a concentration of 100 μM [%].

**Compound**	**84**	**85**	**86**	**87**	**89**	**90**
LNCaP 24h	46.91	90.32	76.71	58.10	81.69	52.96
LNCaP 48h	84.11	83.91	64.97	84.76	98.01	91.53
DU145 24h	83.28	93.98	92.50	78.89	76.11	73.34
DU145 48h	75.12	88.34	65.62	81.46	80.23	79.01
PC3 24h	75.53	65.75	87.66	68.82	66.46	64.11
PC3 48h	75.95	76.40	70.95	70.07	69.08	68.10
BJ 24h	100	118	92	81	101	83
BJ 48h	96	104	106	105	113	105

Table S2. Possible toxic and adverse effects at P_a>=P_i

**P_a**	**P_i**	**Toxic and adverse effects**
0.350	0.093	MAP kinase kinase 6 inhibitor
0.225	0.023	Cyclin-dependent kinase 8 inhibitor
0.227	0.061	MAP kinase kinase 3 inhibitor
0.190	0.045	MAP kinase kinase 5 inhibitor
0.099	0.034	5 Hydroxytryptamine agonist
0.050	0.012	Antiviral (Bovine viral diarrhea virus)
0.043	0.010	CXC chemokine receptor agonist
0.029	0.007	CC chemokine 8 receptor antagonist
0.024	0.006	Histone-lysine N-methyltransferase EZH2 inhibitor
0.025	0.007	Cyclin T1 inhibitor
0.048	0.048	Nicotinic alpha3beta2 receptor antagonist

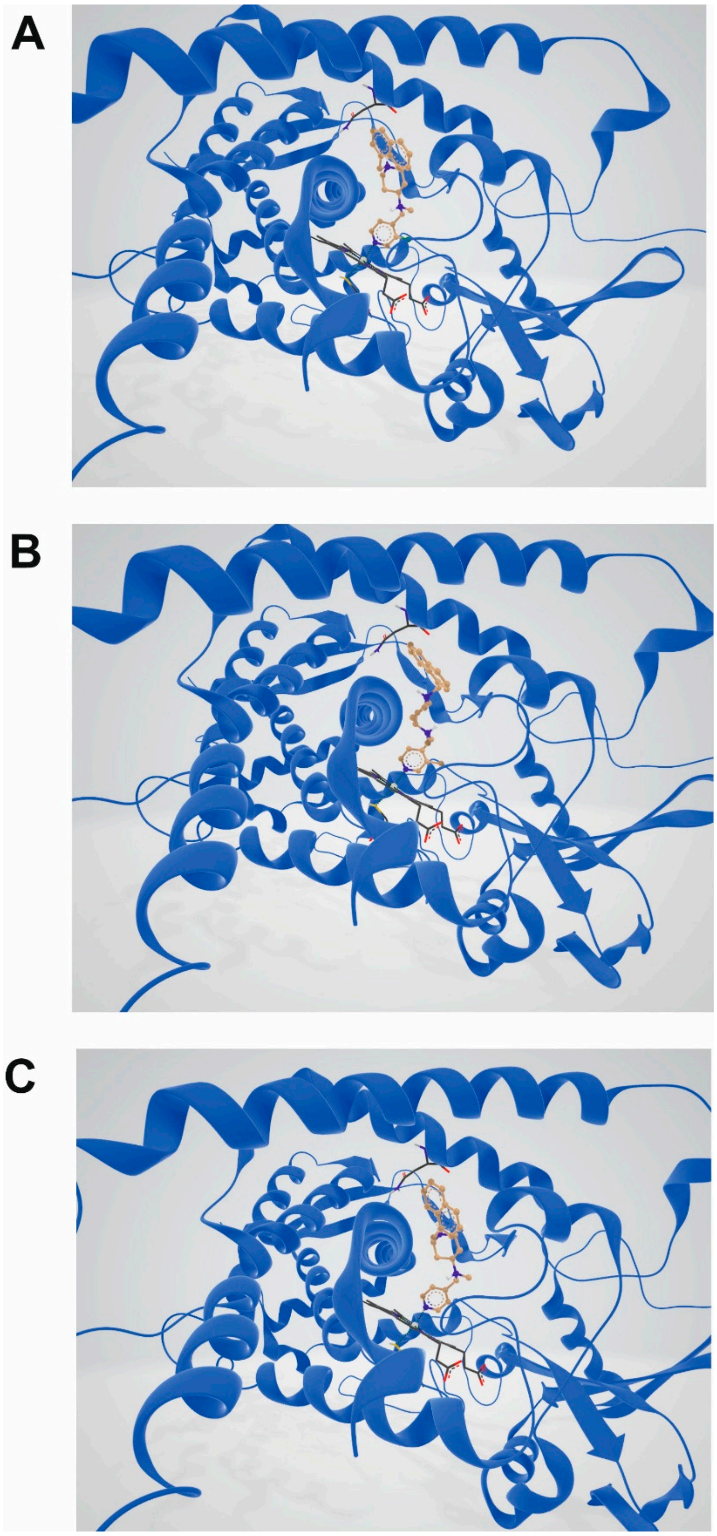

Figure S1. Complexes between the CYP17A1 protein (PDB: 3RUK) and ligands (A) **4a**, (B) **4b**, and (C) **4c**, proposed by SeeSAR. The coordination bond between the heme group and the pyridine nitrogen atom is highlighted, along with two amino acid residues: Asn202 and Cys442. The pocket itself is composed of the following

residues: Ala105, Ala113, Phe114, Ile198, Tyr201, Asn202 (a key residue proximal to the aromatic naphthalene fragment), Glu203, Ile205, Ile206, Leu209, Leu214, His235, Arg239, Gly297, Asp298, Ile299, Phe300, Gly301, Ala302, Gly303, Glu305, Thr306, Val366, Ala367, Leu370, Ile371, Cys442 (the residue coordinating the heme), Val482, and Val483

Molecule 1

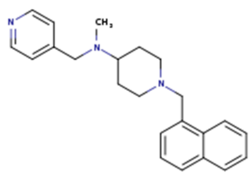

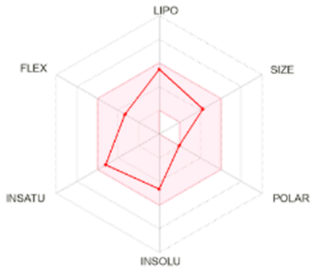

SMILES CN(C1CCN(CC1)Cc1cccc2c1cccc2)Cc1ccncc1

Physicochemical Properties	
Formula	C23H27N3
Molecular weight	345.48 g/mol
Num. heavy atoms	26
Num. arom. heavy atoms	16
Fraction Csp3	0.35
Num. rotatable bonds	5
Num. H-bond acceptors	3
Num. H-bond donors	0
Molar Refractivity	112.44
TPSA	19.37 Å²

Lipophilicity	
Log $P_{o/w}$ (iLOGP)	3.50
Log $P_{o/w}$ (XLOGP3)	4.02
Log $P_{o/w}$ (WLOGP)	3.65
Log $P_{o/w}$ (MLOGP)	3.05
Log $P_{o/w}$ (SILICOS-IT)	4.02
Consensus Log $P_{o/w}$	3.65

Water Solubility	
Log *S* (ESOL)	-4.64
Solubility	7.92e-03 mg/ml ; 2.29e-05 mol/l
Class	Moderately soluble
Log *S* (Ali)	-4.13
Solubility	2.56e-02 mg/ml ; 7.42e-05 mol/l
Class	Moderately soluble
Log *S* (SILICOS-IT)	-7.21
Solubility	2.14e-05 mg/ml ; 6.19e-08 mol/l
Class	Poorly soluble

Pharmacokinetics	
GI absorption	High
BBB permeant	Yes
P-gp substrate	Yes
CYP1A2 inhibitor	No
CYP2C19 inhibitor	No
CYP2C9 inhibitor	No
CYP2D6 inhibitor	Yes
CYP3A4 inhibitor	Yes
Log K_p (skin permeation)	-5.55 cm/s

Druglikeness	
Lipinski	Yes; 0 violation
Ghose	Yes
Veber	Yes
Egan	Yes
Muegge	Yes
Bioavailability Score	0.55

Medicinal Chemistry	
PAINS	0 alert
Brenk	0 alert
Leadlikeness	No; 1 violation: XLOGP3>3.5
Synthetic accessibility	2.20

Figure S2. Modeled ADME physicochemical data (full SwissADME report)

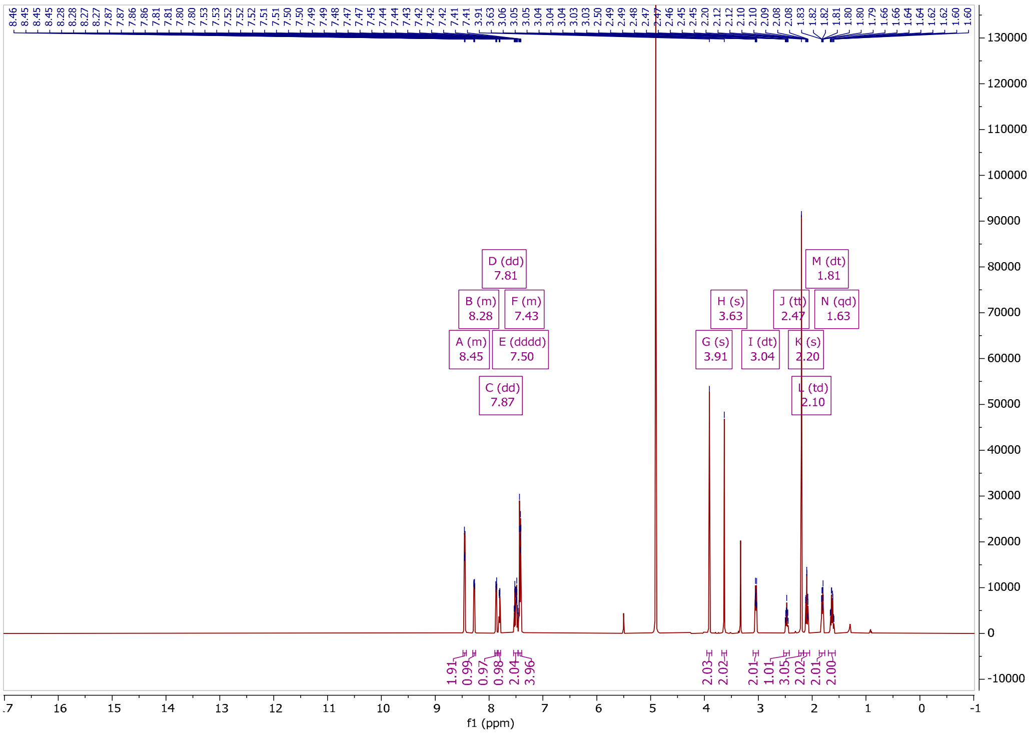

Figure S3. ^{1}H NMR spectrum of compound **4a**.

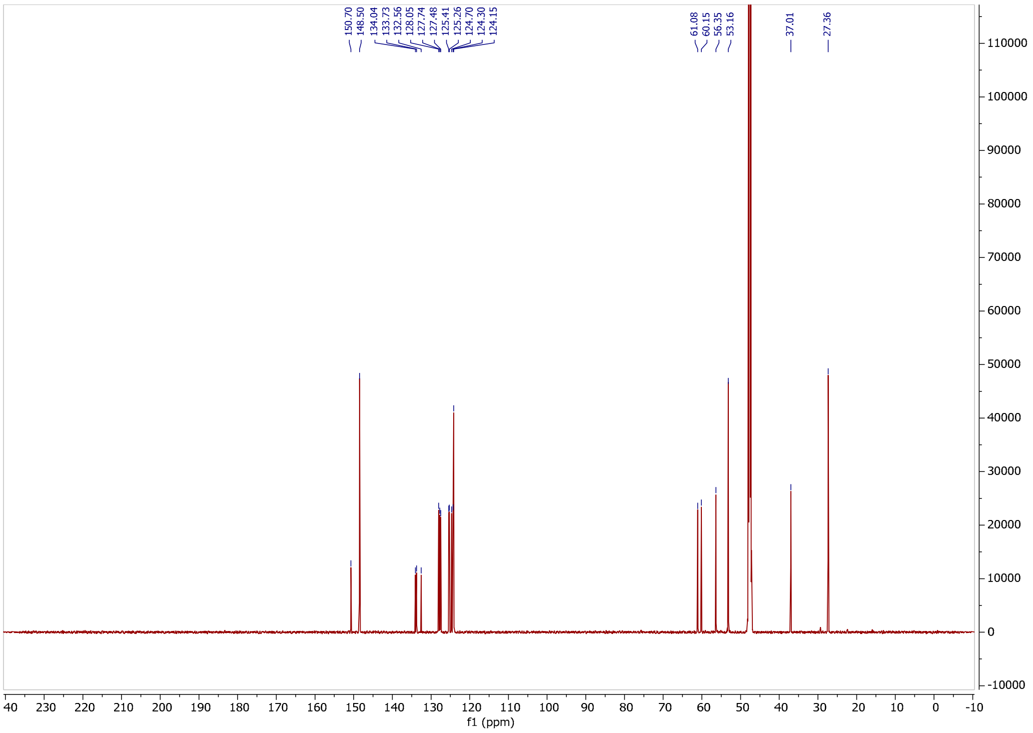

Figure S4. ^{13}C NMR spectrum of compound **4a**.

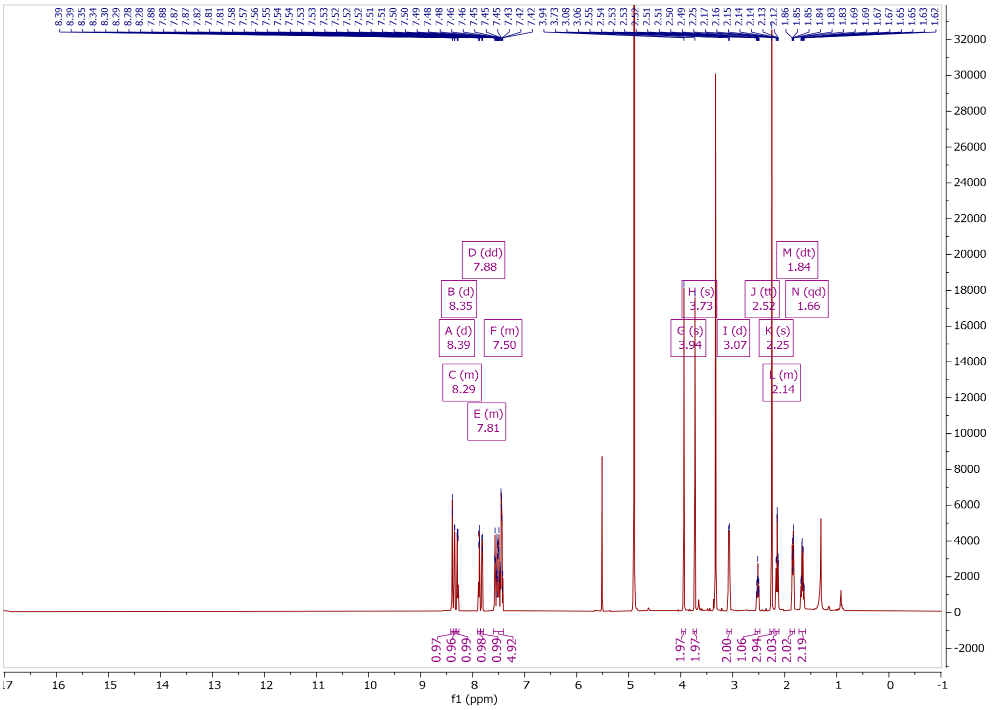

Figure S5. ^{1}H NMR spectrum of compound **4b**.

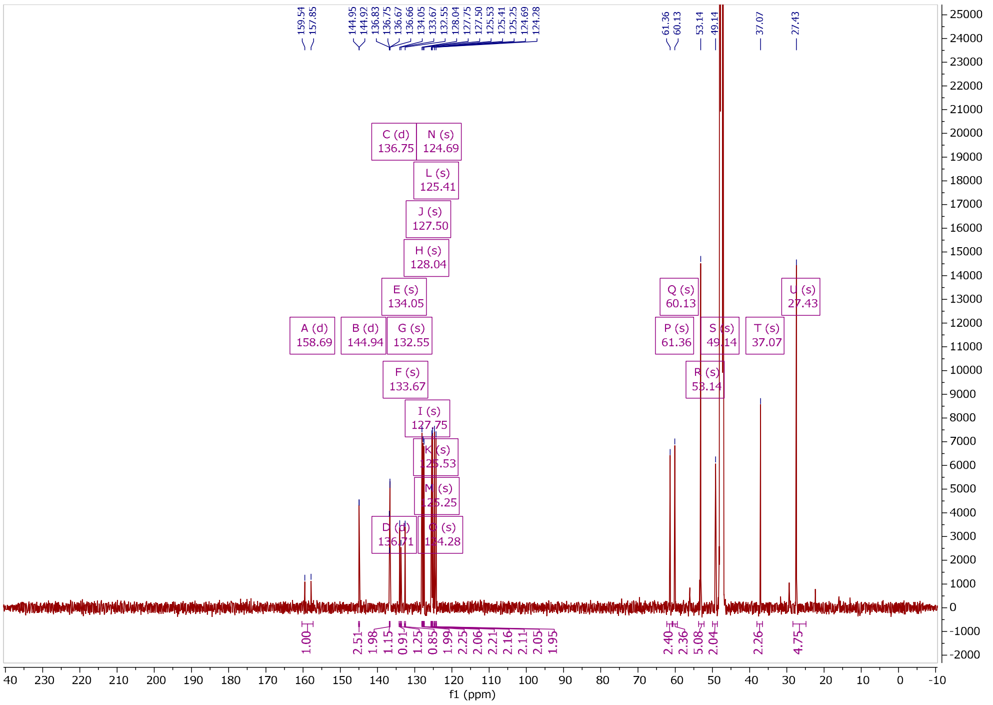

Figure S6. ^{13}C NMR spectrum of compound **4b**.

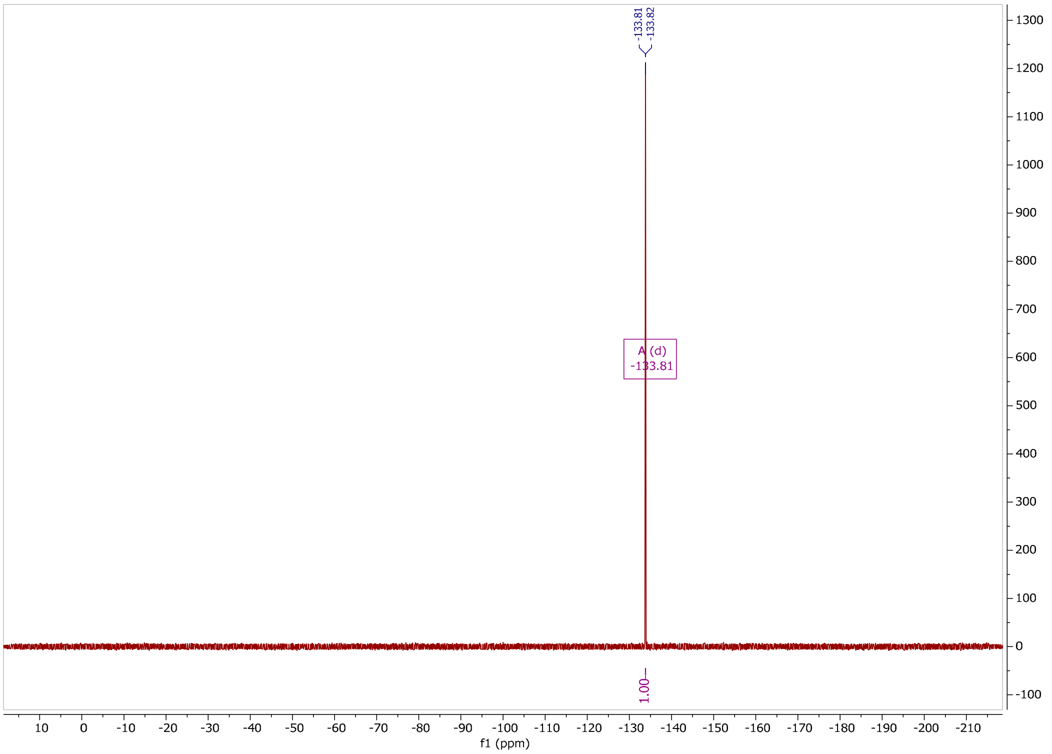

Figure S7. ^{19}F NMR spectrum of compound **4b**.

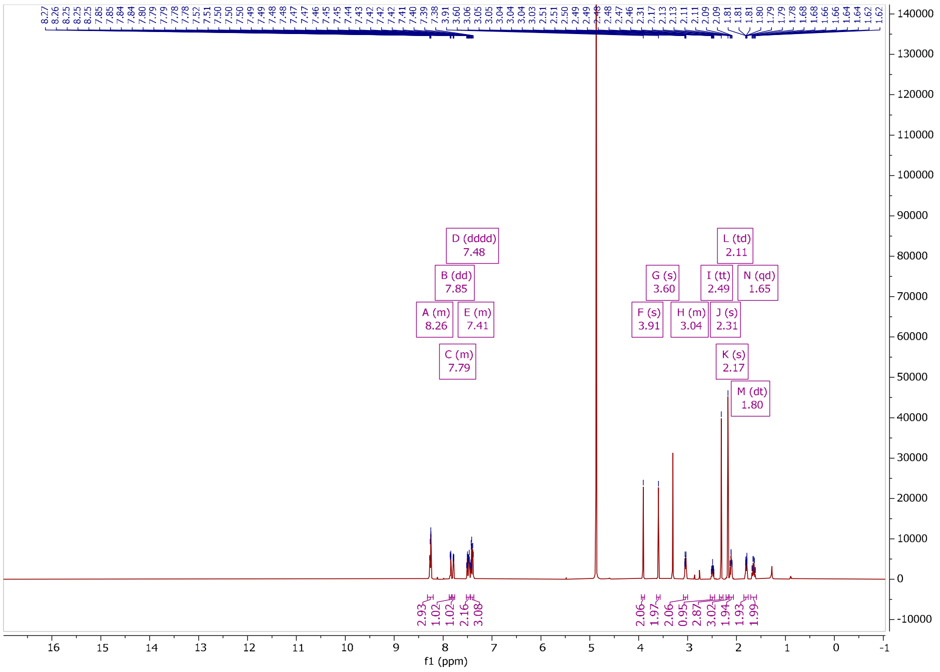

Figure S8. ^{1}H NMR spectrum of compound **4c**.

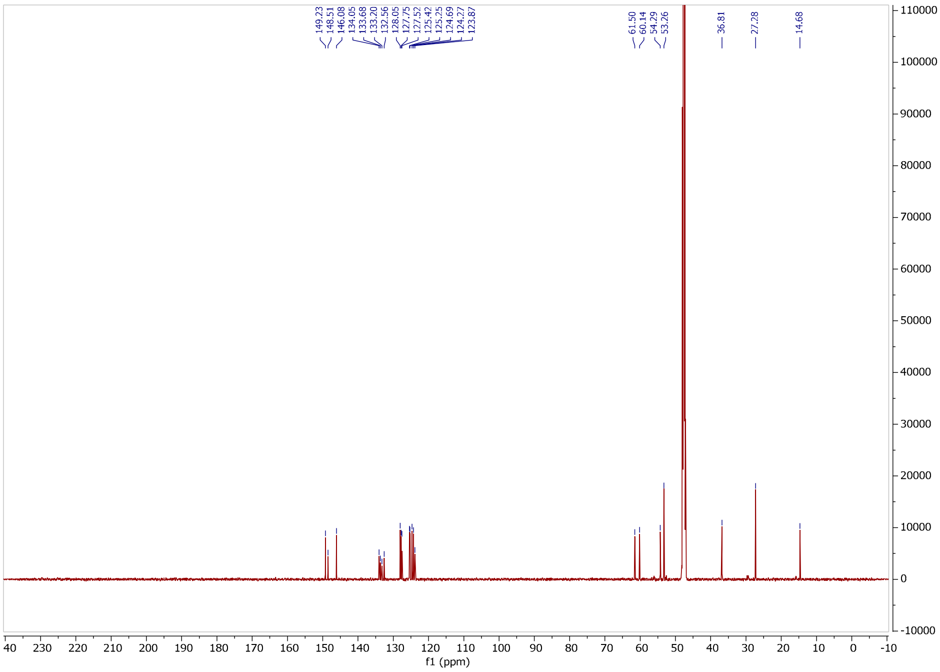

Figure S9. ^{13}C NMR spectrum of compound **4c**.

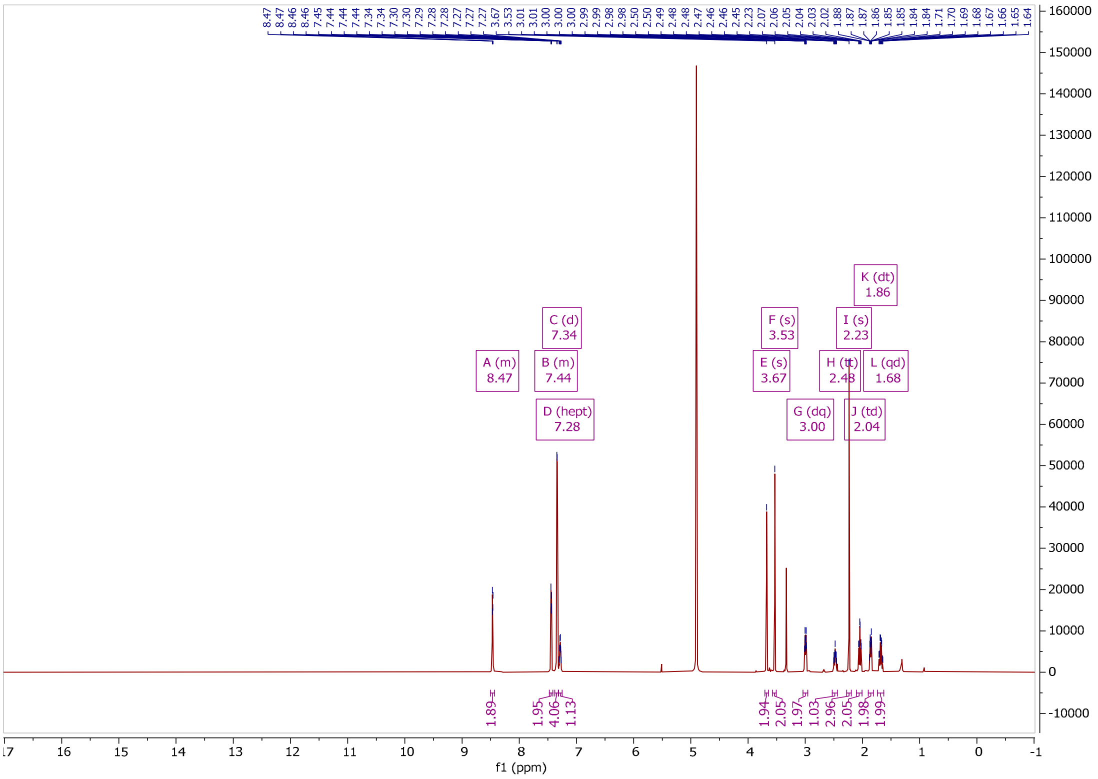

Figure S10. ^{1}H NMR spectrum of compound **4d**.

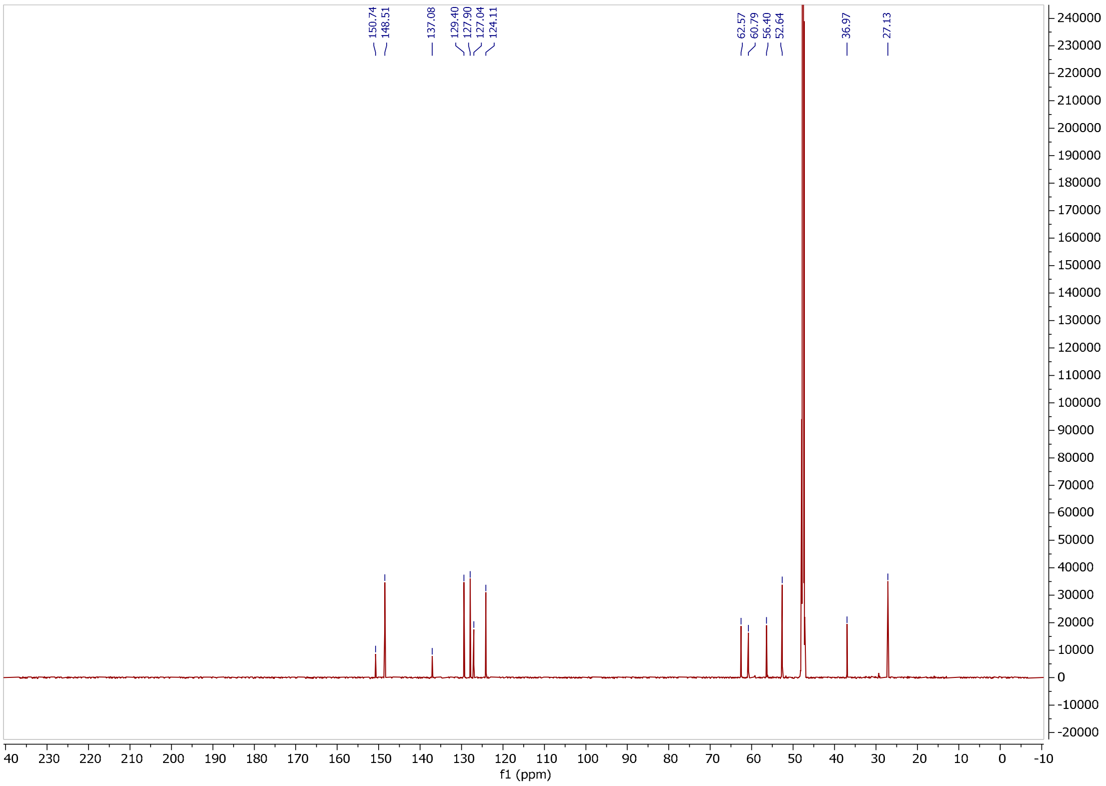

Figure S11. ^{13}C NMR spectrum of compound **4d**.

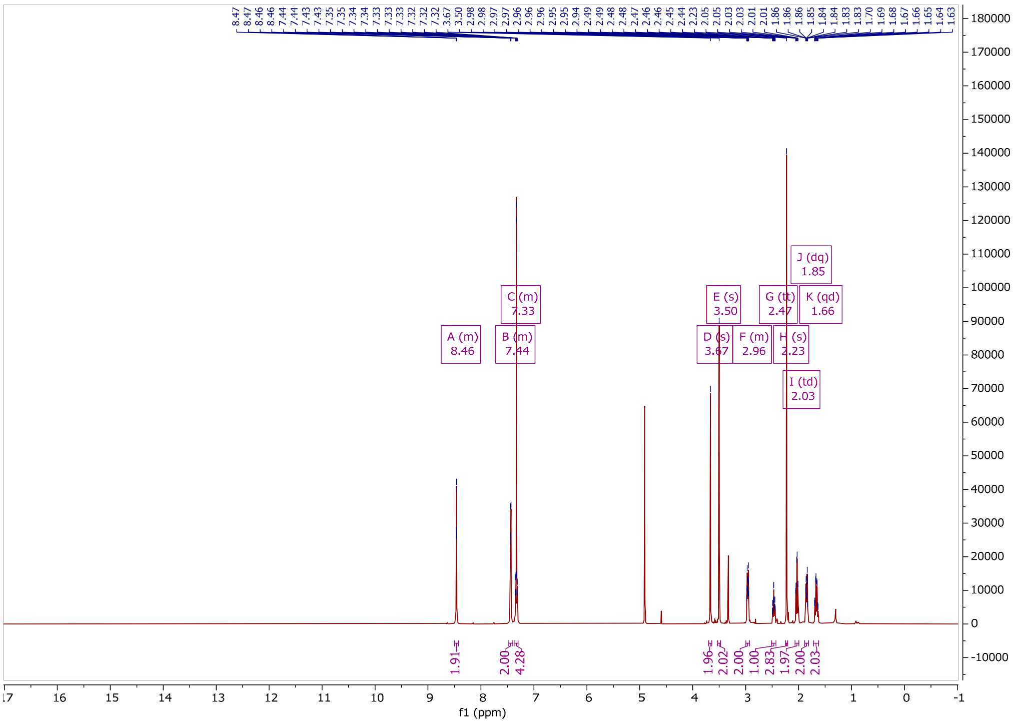

Figure S12. ^{1}H NMR spectrum of compound **4e**.

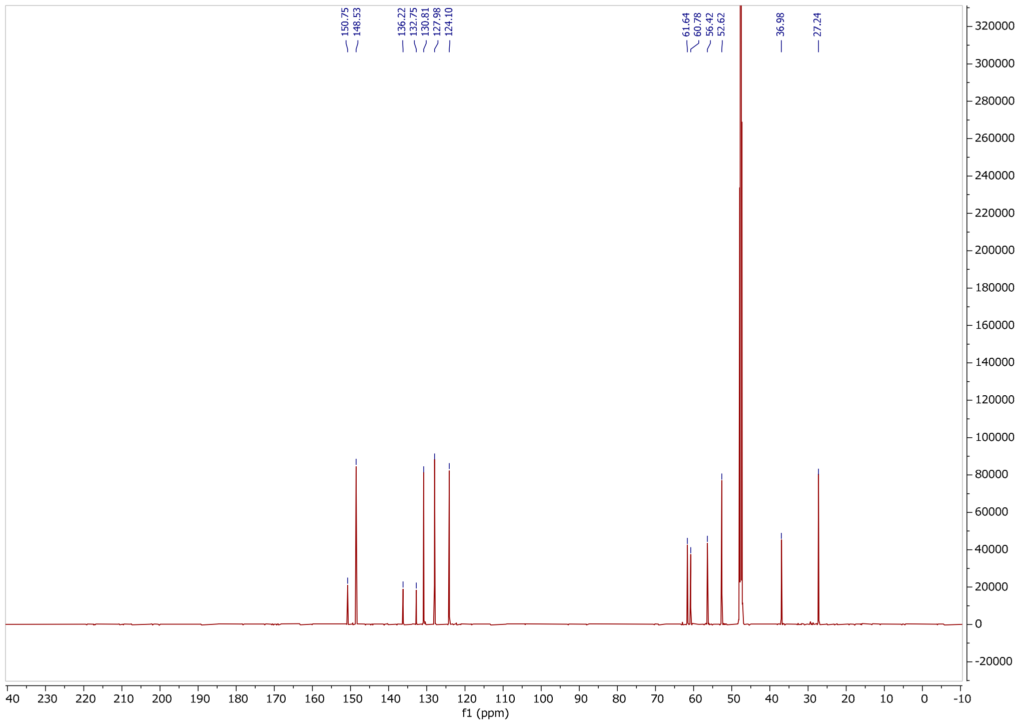

Figure S13. ^{13}C NMR spectrum of compound **4e**.

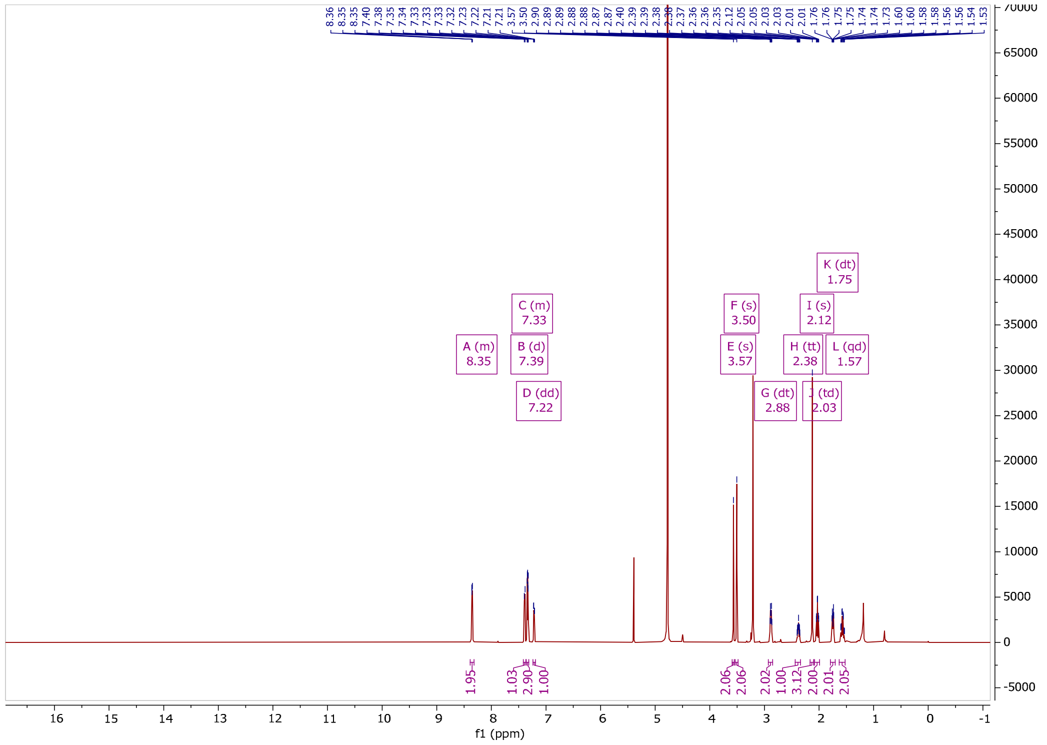

Figure S14. ^{1}H NMR spectrum of compound **4f**.

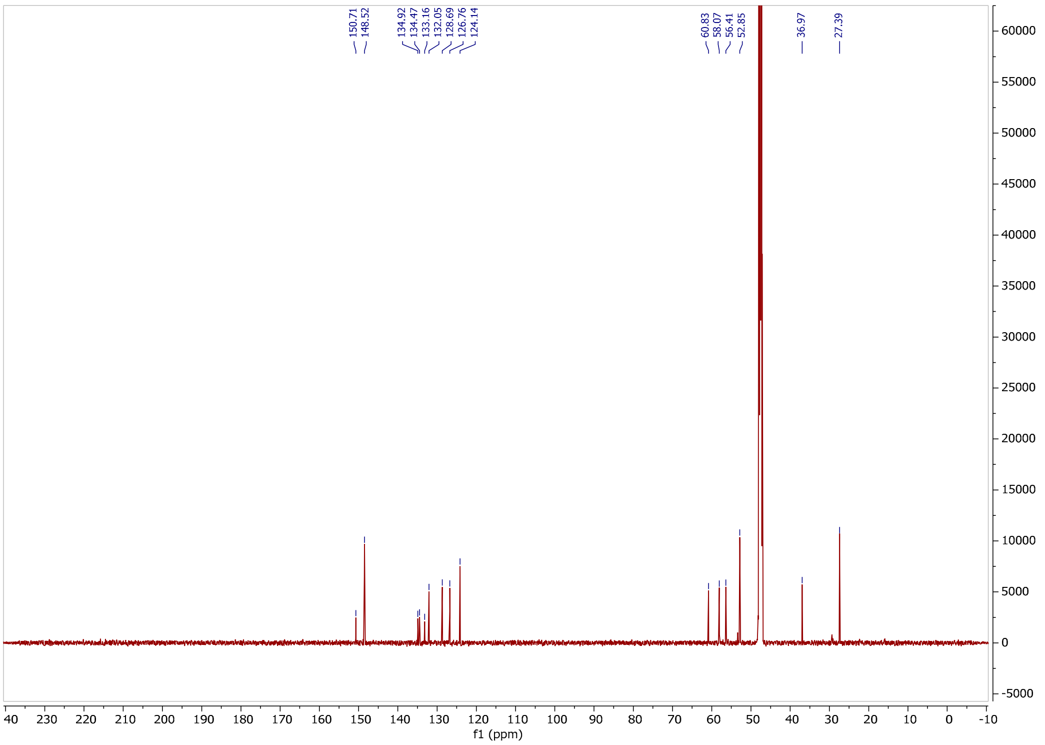

Figure S15. ^{13}C NMR spectrum of compound **4f**.

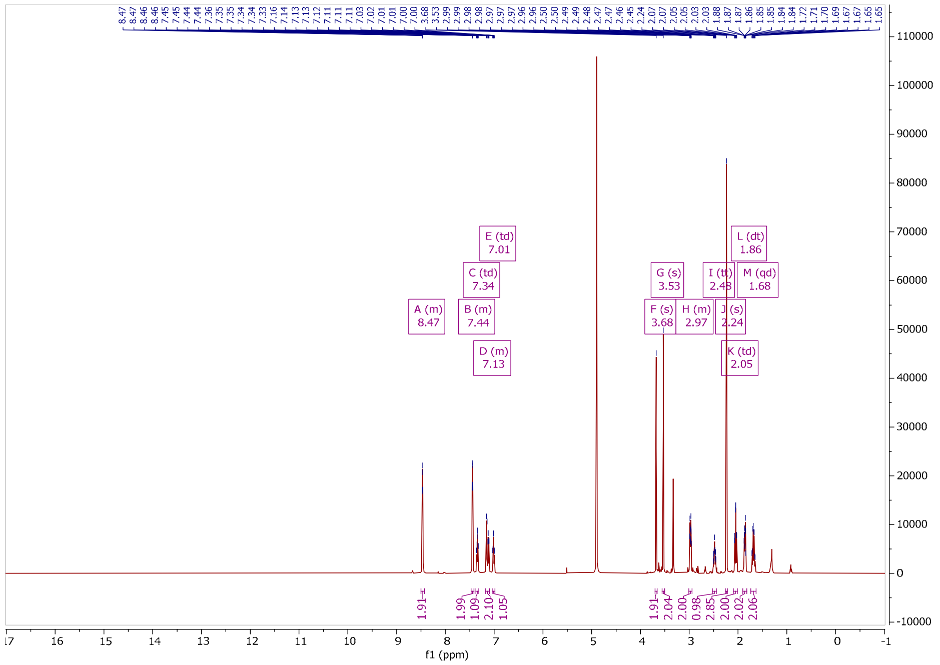

Figure S16. ^{1}H NMR spectrum of compound **4g**.

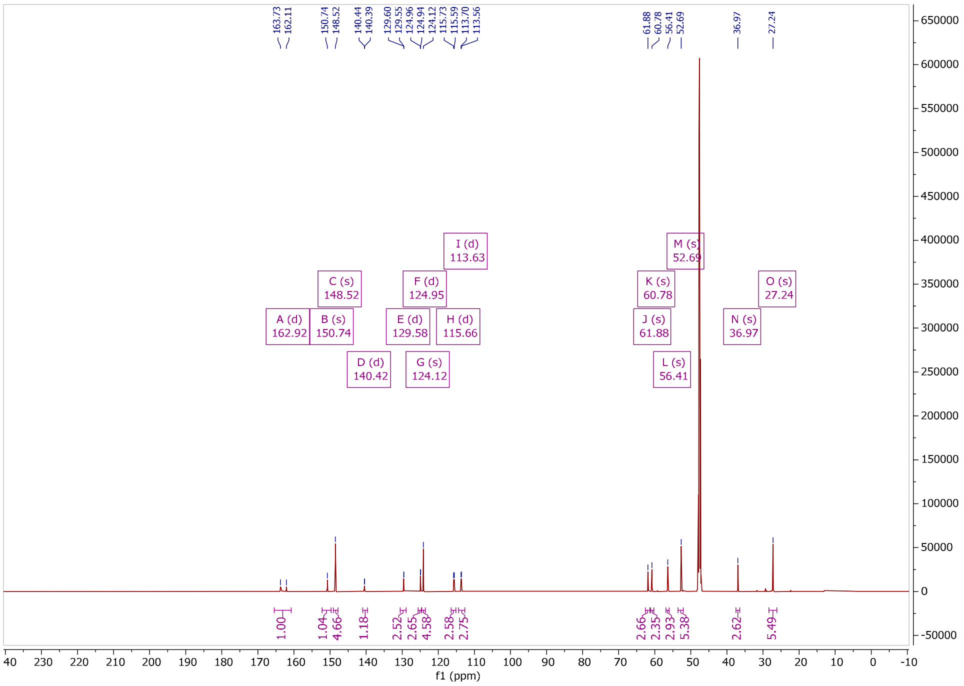

Figure S17. ^{13}C NMR spectrum of compound **4g**.

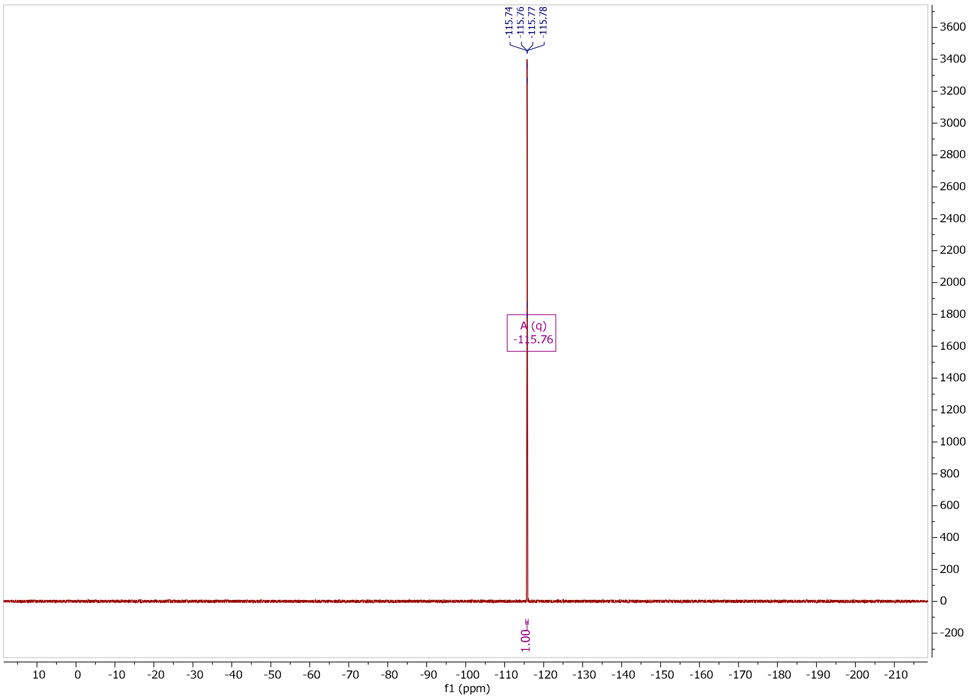

Figure S18. ^{19}F NMR spectrum of compound **4g**.

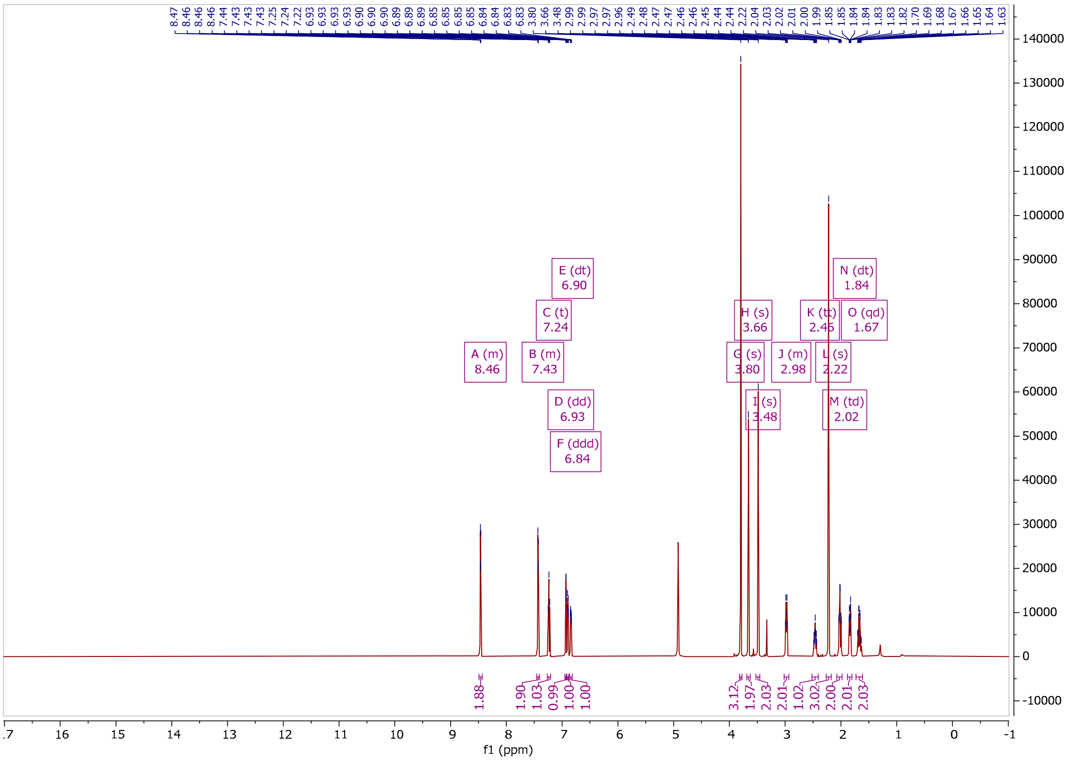

Figure S19. ^{1}H NMR spectrum of compound **4h**.

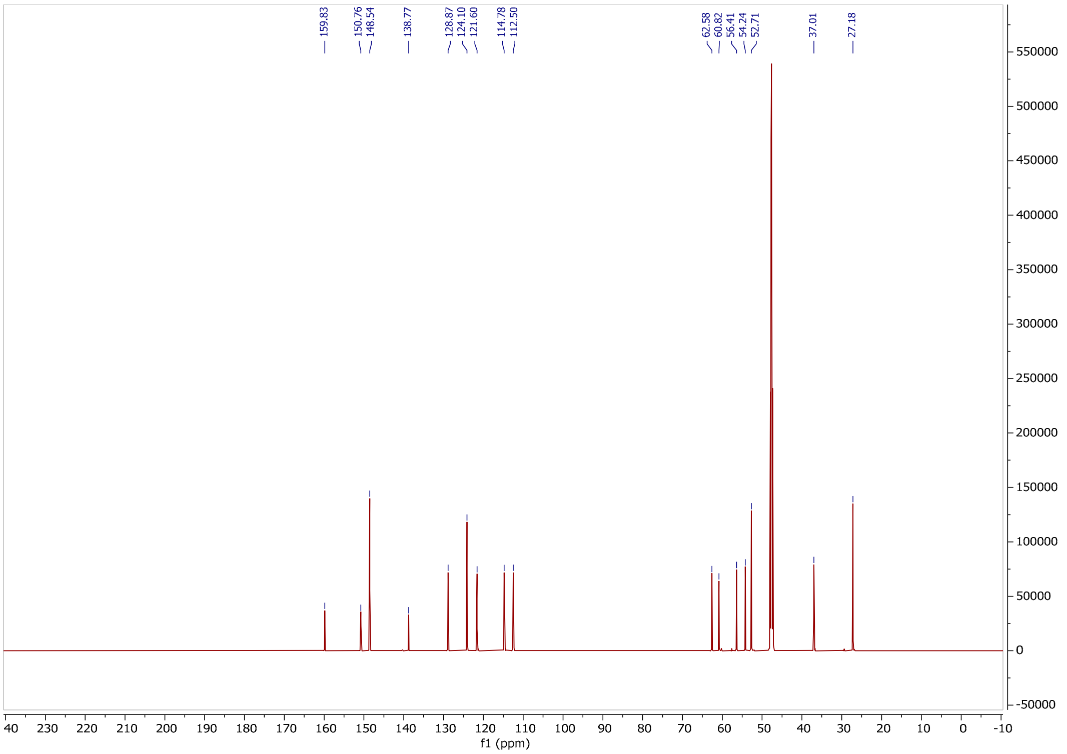

Figure S20. ^{13}C NMR spectrum of compound **4h**.

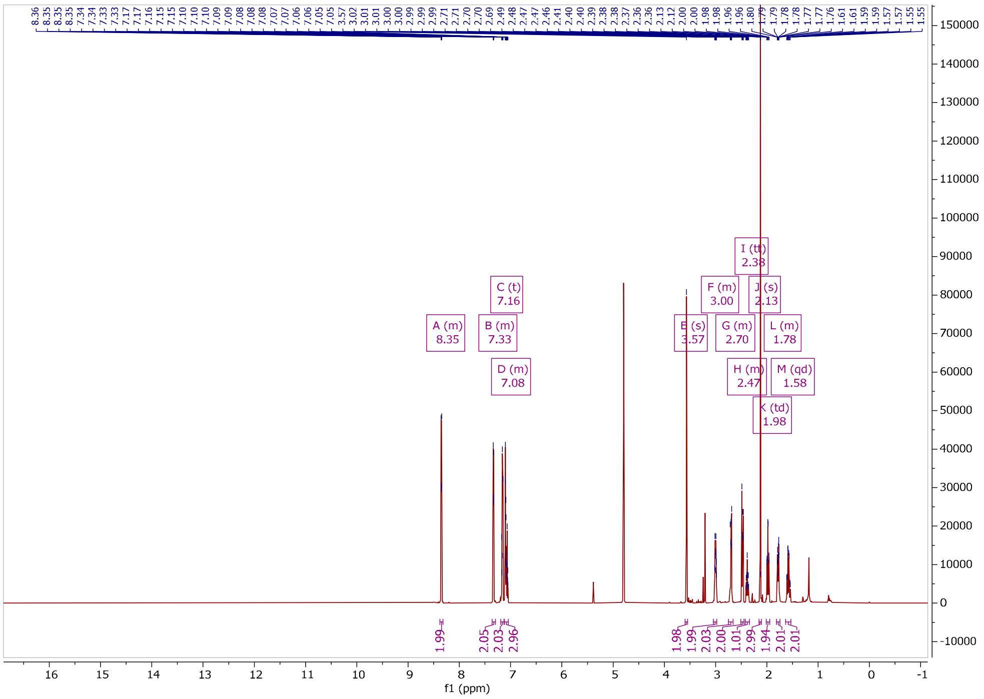

Figure S21. ^{1}H NMR spectrum of compound **4i**.

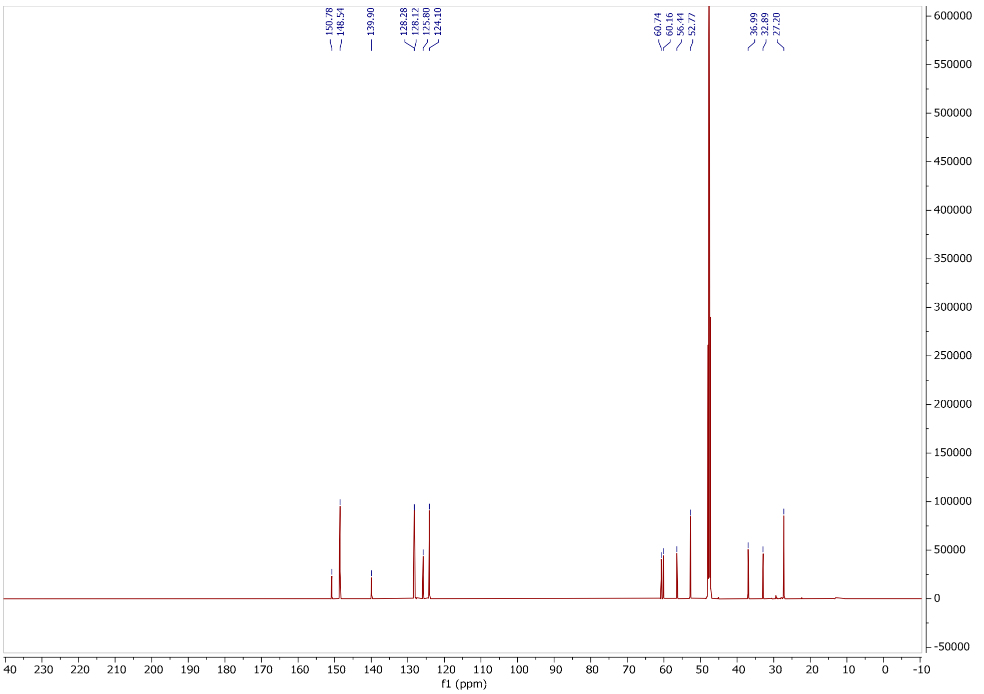

Figure S22. ^{13}C NMR spectrum of compound **4i**.

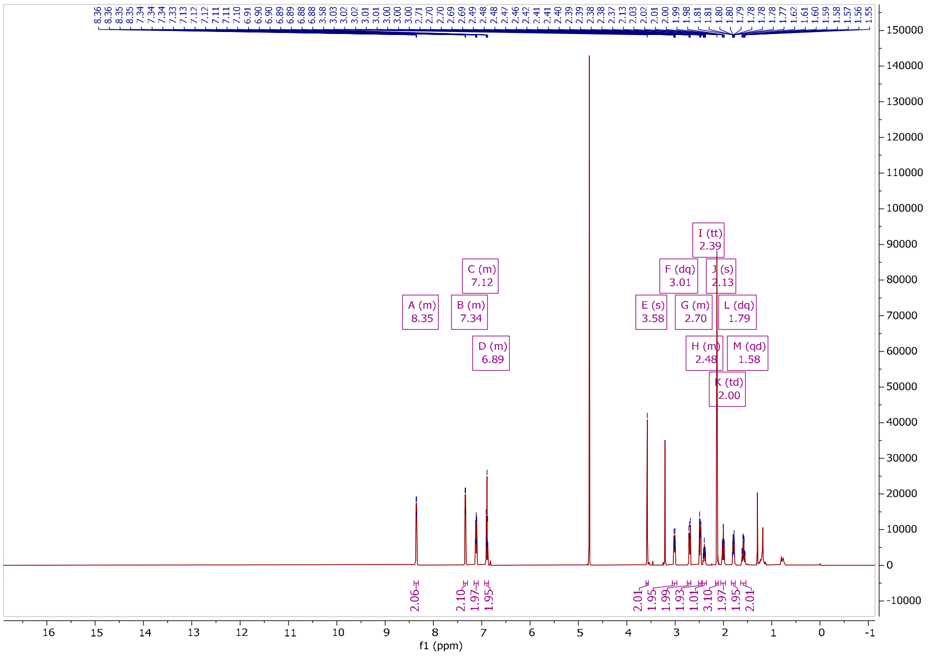

Figure S23. ^{1}H NMR spectrum of compound **4j**.

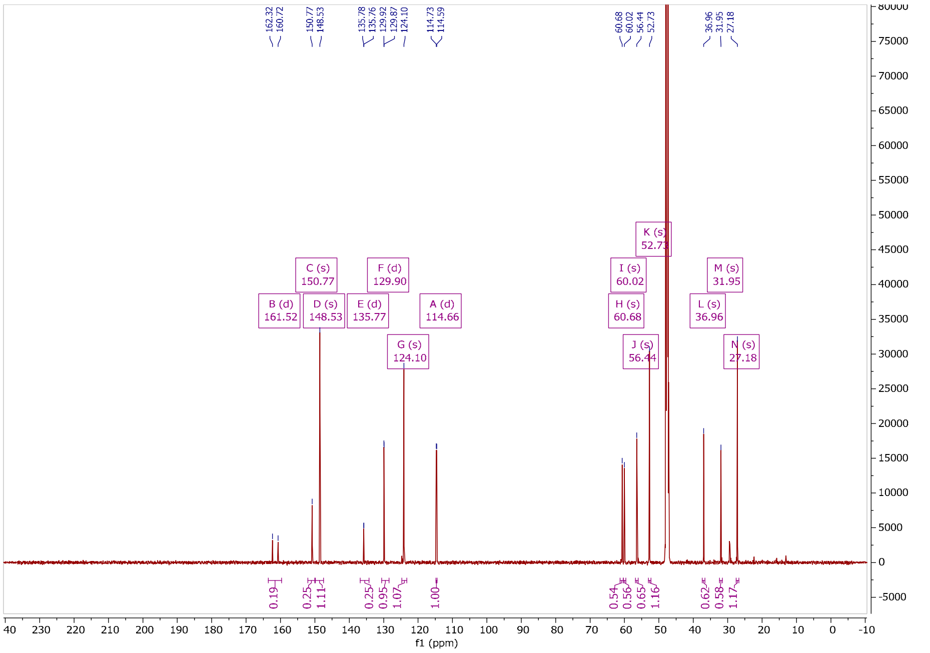

Figure S24. ^{13}C NMR spectrum of compound **4j**.

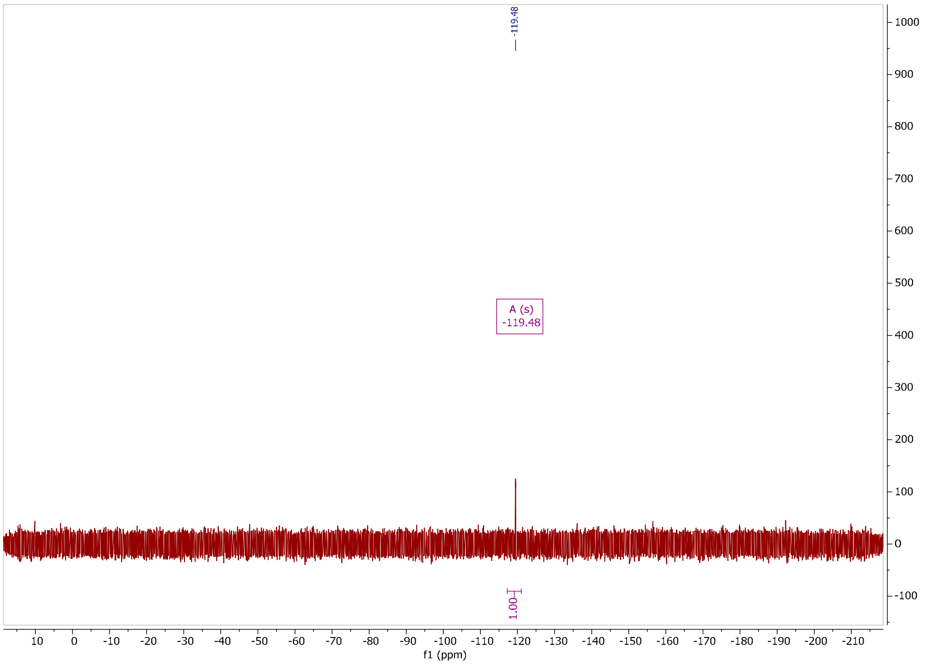

Figure S25. ^{19}F NMR spectrum of compound **4j**.

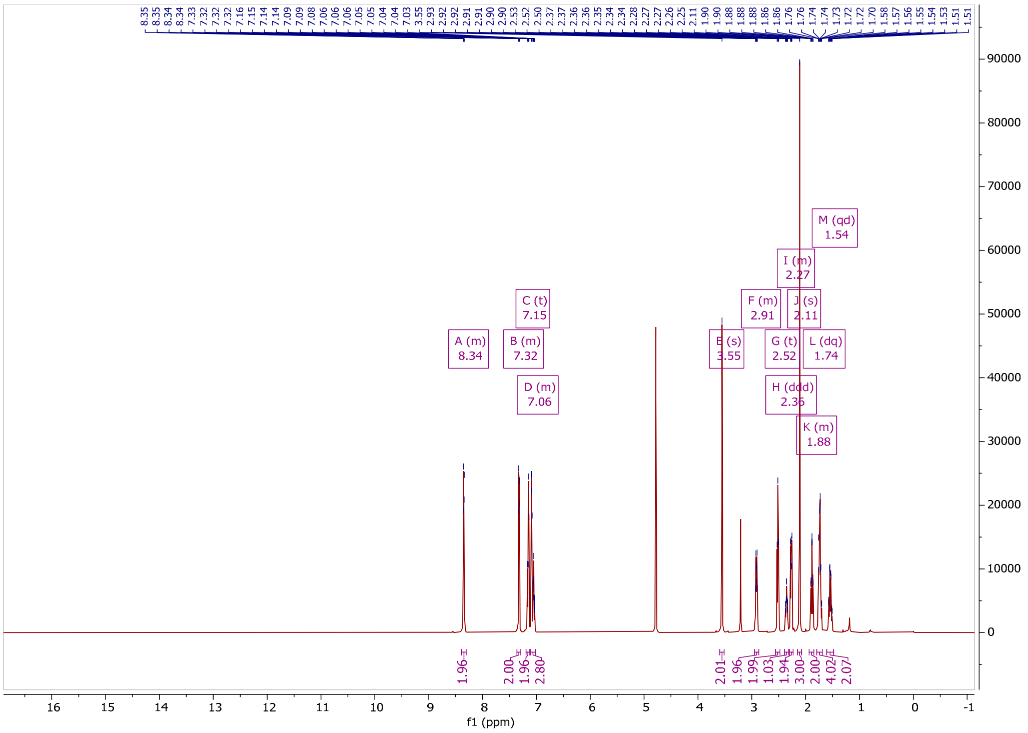

Figure S26. ^{1}H NMR spectrum of compound **4k**.

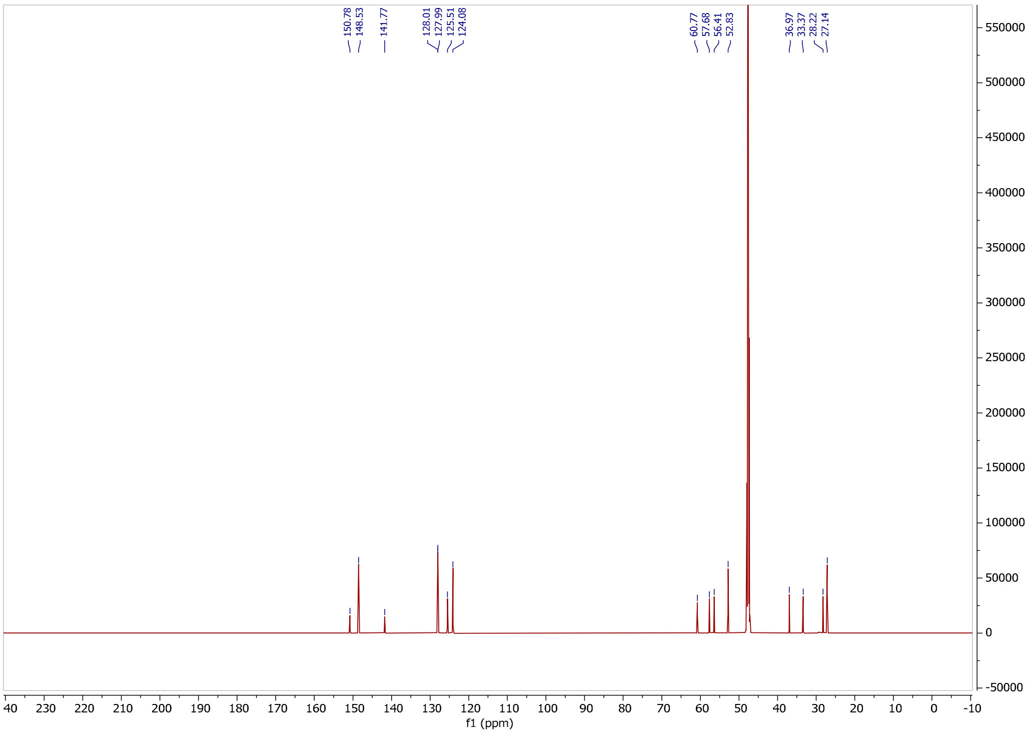

Figure S27. ^{13}C NMR spectrum of compound **4k**.

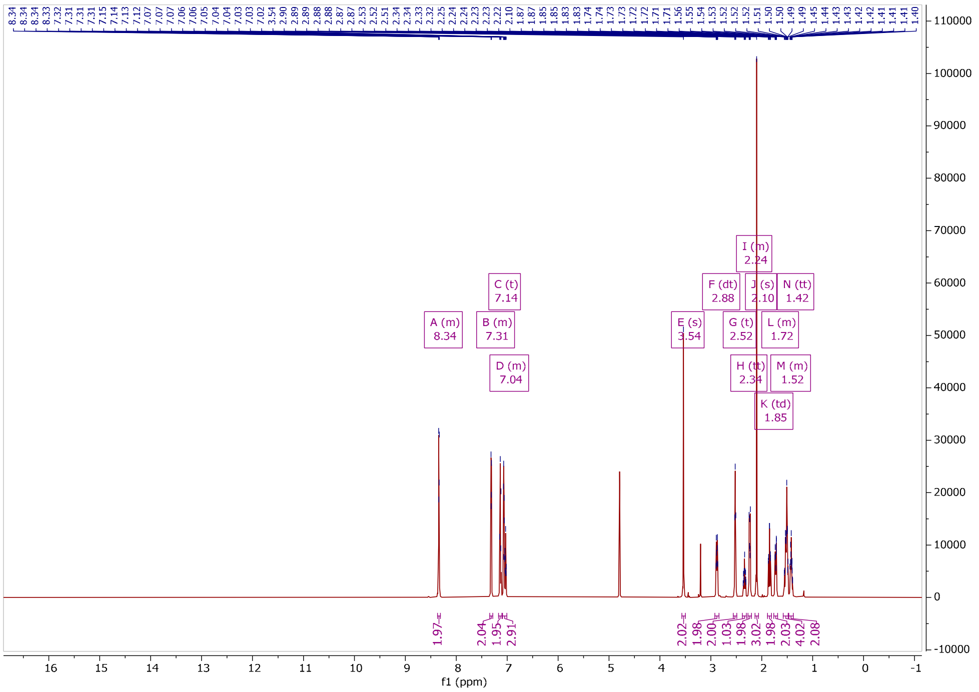

Figure S28. ^{1}H NMR spectrum of compound **4l**.

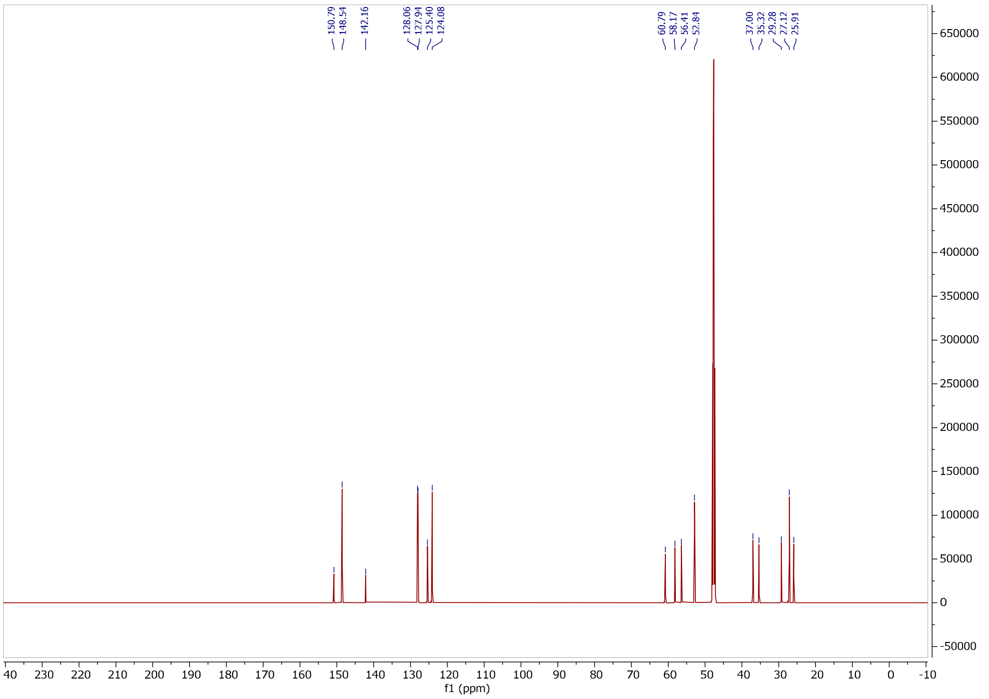

Figure S29. ^{13}C NMR spectrum of compound **4l**.

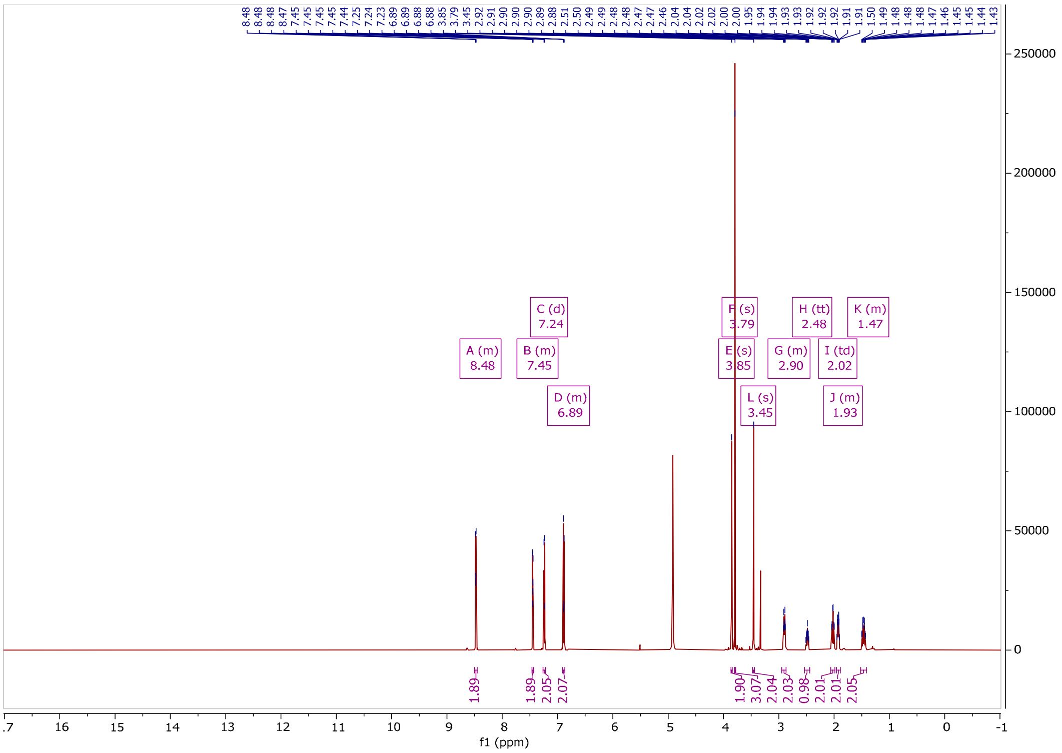

Figure S30. ^{1}H NMR spectrum of compound **4m**.

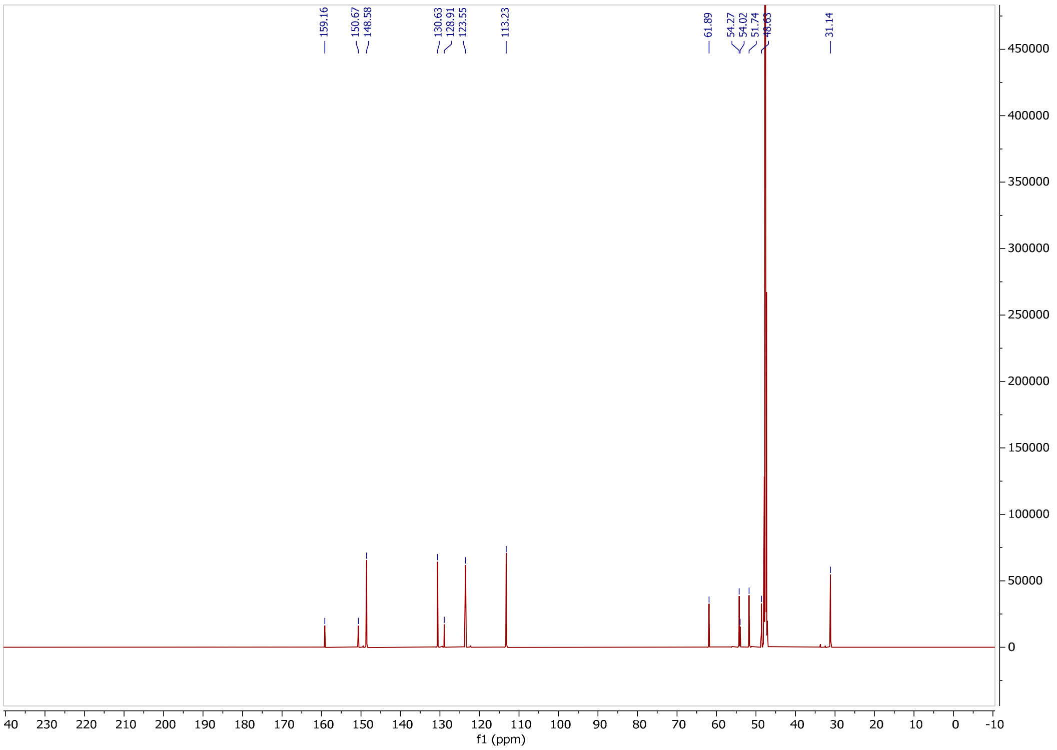

Figure S31. ^{13}C NMR spectrum of compound **4m**.
